# Hydrophilicity-Enhanced NH_2_‑MIL-88B(Fe) Integrated Photocatalytic Membrane Reactor for Simultaneous Rejection and Degradation of Low-Density Polyethylene in Water Matrices

**DOI:** 10.1021/acsami.5c21187

**Published:** 2026-03-16

**Authors:** Guan-Yu Chen, Yu-Lin Chen, Kazuki Harada, Masaaki Yoshida, Qiang Lyu, Chia-Her Lin, Li-Chiang Lin, Chechia Hu

**Affiliations:** a Department of Chemical Engineering, Sustainable Electrochemical Energy Development Center (SEED), 34878 National Taiwan University of Science and Technology, Daan Dist., Taipei City 106335, Taiwan; b Applied Chemistry, Graduate School of Sciences and Technology for Innovation, 13150 Yamaguchi University, Ube, Yamaguchi 755-8611, Japan; c Department of Materials Physics, China University of Petroleum (East China), Qingdao, Shandong 266580,China; d Department of Chemistry, National Tsing Hua University, Hsinchu City 300044, Taiwan; e Department of Chemical Engineering, 33561 National Taiwan University, Daan Dist Taipei City 106335, Taiwan

**Keywords:** microplastic removal, photocatalytic membrane reactor, wastewater treatment, amine modification, photocatalytic degradation

## Abstract

Microplastic (MP) plastic particles with diameters of up to 5 mm have emerged as pervasive global pollutants, posing serious risks to both human and environmental health. In this study, a photocatalytic membrane reactor (PMR) was designed to perform dual functions of filtration and photocatalytic decomposition of low-density polyethylene (LDPE) particles. An NH_2_-modified MIL-88B­(Fe) (NM88B) photocatalyst was prepared and then used as a precursor to grow a photocatalyst membrane (MRS-NM88B) on an alumina membrane via the modified reactive seeding (MRS) method. Compared with unmodified MIL-88B­(Fe) (M88B), NM88B exhibited higher hydrophilicity with enhanced permeance flux and better photocatalytic performance. The PMR fabricated using the MRS-NM88B membrane successfully rejected and degraded LDPE particles, demonstrating a high LDPE particle removal efficiency of 96.9% under dark conditions and a 22.3% mass loss of LDPE particles under light irradiation. Furthermore, the PMR can be applied to real river water for effective filtration and degradation. The results demonstrate the potential of this PMR for microplastic removal and degradation in wastewater treatment applications.

## Graphical abstract

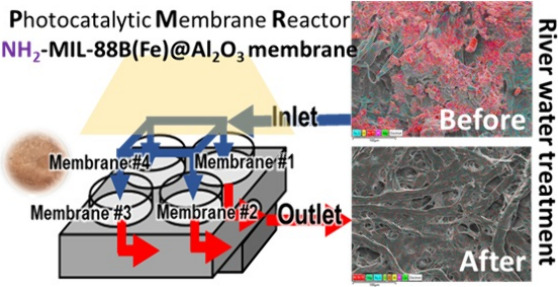

## Introduction

The global annual plastic production is approximately 150 million tons,^1^ with only <5% being recycled,^2^ resulting in a huge amount of waste. It is predicted that the accumulated plastic in the environment will reach 155 to 265 million tons by 2060,^3,4^ leading to severe environmental pollution problems. Plastic waste accumulates in rivers, oceans, different water matrices, and landfills, severely damaging the quality of water, soil, and air, and posing a serious threat to ecosystems.^5,6^ In particular, plastic particles smaller than 5 mm, the so-called microplastics,^7^ have become hazardous pollutants and have been the subject of intense research in terms of their health and environmental impacts since the 1980s and 1990s.^8^ Microplastics are composed of common polymers such as polyethylene, polyacrylates, polyamides, nylon, polyesters, polypropylene, polystyrene, and polyurethane,^9^ as well as some special industrial polymers, with polyethylene being the most prevalent.^10^

Microplastics can be removed from water using filtration-based or degradation-based approaches, among which filtration-based separation techniques are considered one of the most effective methods for an almost complete separation of microplastics.^11–15^ Advanced wastewater treatment technologies installed in wastewater treatment plants, such as membrane bioreactors, rapid sand filtration, flotation, and disc filters, can substantially reduce the discharge of microplastics into aquatic environments.^16,17^ However, most filtration or separation processes face challenges such as membrane fouling and reduced efficiency for submicron or nanoplastic particles, which limit their long-term stability and practical scalability. Therefore, integrating light-driven degradation into water treatment systems has attracted increasing attention, as photocatalytic processes can not only prevent membrane fouling but also decompose microplastics into smaller or mineralized products, offering a sustainable solution beyond physical separation. Despite its potential, research on microplastic photodegradation remains currently at the laboratory stage, focusing on the fabrication of films from photocatalysts and plastics, the preparation of buffer solutions with plastic particles and photocatalysts, and the development of photodegradation reactions.^18,19^ For instance, photodegradation has been conducted on composite films of LDPE and photocatalysts, such as TiO_2_ or g-C_3_N_4_,^20,21^ and on mixtures of microplastic particles of high-density polyethylene and photocatalyst particles in a beaker or a lab-scale reactor.^22,23^

Among various photocatalysts, metal–organic frameworks (MOFs) stand out owing to their ultrahigh porosity, tunable pore sizes, and large specific surface areas, which stem from their crystalline and porous periodic network structures formed by the self-assembly of metal ions or metal clusters and organic ligands. In addition to photocatalysis, MOFs have enormous potential in applications such as antibacterial agents, gas storage, adsorption and separation, and sensing.^24–28^ In the synthesis of MOFs, using organic linkers bearing amino (−NH_2_) groups can lead to materials with enhanced photocatalytic properties and photostability. These −NH_2_ linkers also possess strong electron transfer capabilities, which can reduce the bandgap of the materials.^29^ The −NH_2_ functionalities can also form strong hydrogen bonds with water molecules, thereby increasing the polarity of the material and its affinity toward aqueous environments, improving water adsorption, and facilitating interactions with polar solvents or biomolecules. For example, Zhang et al.^30^ synthesized MIL-101-NH_2_ by replacing terephthalic acid (H_2_BDC) with 2-aminoterephthalic acid (NH_2_BDC). It was found that the −NH_2_ groups can absorb visible light and transfer electrons to the Fe–O clusters in MIL-101-NH_2_, thereby generating •OH and •SO_4_^–^ radicals under visible light for the degradation of amaranth. Xie et al.^31^ prepared poly­(vinylidene fluoride)/NH_2_-MIL-88B­(Fe) membranes using a phase transition method. The addition of NH_2_-MIL-88B­(Fe) substantially improved the surface hydrophilicity, film flux, photocatalytic degradation resistance to fouling, and absorption capacity for visible light, achieving a 97% degradation rate for methylene blue (MB) within 70 min. Shi et al.^32^ also prepared NH_2_-MIL-88B­(Fe) using a microwave-assisted solvothermal method, showing that under visible-light irradiation, the NH_2_-functionalized organic linkers and Fe–O clusters in NH_2_-MIL-88B­(Fe) were excited, and the photogenerated electrons were transferred to the Fe–O clusters. These studies revealed that NH_2_-MIL-88B­(Fe) exhibited higher photocatalytic activity than MIL-88B­(Fe), demonstrating that the photocatalytic efficiency of MOFs can be adjusted by simply modifying the organic linkers in the MOF structure. Overall, owing to their large surface area, tunable porosity, and abundant active sites, MOFs have shown great potential as photocatalysts for environmental remediation. Despite these advantages, most MOFs are synthesized in powder form, making their direct use in aqueous systems impractical due to poor dispersibility and difficulties in catalyst recovery. Therefore, developing MOF-coated support systems is essential, as this integration not only facilitates easy recovery and reuse but also enhances the structural stability of the MOF under continuous operation in water.

Photocatalytic membrane reactors (PMRs) have emerged as a promising integrated catalyst–support system in wastewater treatment, as they enable the simultaneous separation, adsorption, and photodegradation of contaminants through photocatalytic films. PMRs are commonly used to treat homogeneous pollutants such as antibiotics (e.g., tetracycline and sulfadiazine)­[,^33–35^ organic compounds (e.g., humic acid),^36,37^ and dyes.^38^ We aimed to expand the application scope of PMRs by developing a flow system reactor for the simultaneous photodegradation and filtration of microplastics.^39^ To the best of our knowledge, this is the first report on the degradation and filtration of LDPE from different water matrices using a PMR.

In this study, photocatalytic membranes were prepared using the modified reactive seeding (MRS) method, which was previously utilized in our group to fabricate an MRS-MIL-88B­(Fe) membrane containing the MIL-88B­(Fe) photocatalyst. First, an NH_2_-MIL-88B­(Fe) membrane was prepared via −NH_2_ modification. Subsequently, this membrane was used as a precursor to grow an MRS-NH_2_-MIL-88B­(Fe) photocatalyst membrane on top of an alumina membrane via the MRS method. The fabricated membranes were then introduced into a PMR to filter and photodegrade LDPE particles. The repeatability and stability of the PMR in processing LDPE particles were evaluated.

## Experimental Section

### Materials

Terephthalic acid (H_2_BDC, 99.0%) and 2-aminoterephthalic acid (NH_2_BDC, 99.0%) were obtained from ACROS. Iron­(III) chloride (FeCl_3_, anhydrous, 98.0%) and polyethylene powder (LDPE, 98.0–98.8%) were purchased from Alfa Aesar. *N*,*N*-Dimethyl-formamide (DMF, 99.5%) was obtained from DUKSAN. Alumina membranes (diameter = ∼4.5 cm, porosity is 49%) were obtained from Kinik. River water was collected from the Keelung River in Taipei, Taiwan, and used without any purification or pretreatment.

### Fabrication of MIL-88B­(Fe) and NH_2_-MIL-88B­(Fe) Precursor Solutions

For the preparation of the MIL-88B­(Fe) precursor solution, FeCl_3_ (0.811 g, 5 mmol), H_2_BDC (0.83 g, 5 mmol), and DMF (25 mL) were added to a 250 mL beaker and thoroughly mixed via ultrasonic treatment for 25 min. The preparation of the NH_2_-MIL-88B­(Fe) precursor solution was similar, except that NH_2_BDC (0.91 g, 5 mmol) was used as a precursor instead of H_2_BDC. The resulting MIL-88B­(Fe) and NH_2_-MIL-88B­(Fe) samples were named as M88B and NM88B, respectively.

### Fabrication of MRS-MIL-88B­(Fe) and MRS-NH_2_-MIL-88B­(Fe) Membranes

The photocatalyst membranes were fabricated using the MRS method.^40^ Briefly, H_2_BDC (1.66 g, 10 mmol) and DMF (50 mL) were added to a 250 mL beaker and stirred. An alumina membrane was submerged in the solution for 12 h and then dried to obtain the seed layer. The alumina membrane with the seed layer was placed in a 250 mL autoclave, and NM88B and M88B precursor solutions were respectively added. The setup was then placed in an oven for hydrothermal treatment at 150 °C for 15 h. After cooling, the membranes were washed several times with DMF, placed in a DMF solution for 1 h to remove excess unreacted reagents, and then immersed in methanol for another 6 h. Finally, the membranes were dried in an oven at 50 °C. The as-prepared NH_2_-containing (MRS-NH_2_-MIL-88B­(Fe)) and NH_2_-free (MRS-MIL-88B­(Fe)) photocatalyst membranes were named as MRS-NM88B and MRS-M88B, respectively.

### LDPE Particle Removal Using the PMR

Figure S1a (Supporting Information) shows a schematic of the PMR, which can simultaneously accommodate four membranes at position E. In the 3 L tank shown in position B (Figure S1a), 25 mg of LDPE particles and 1 L of deionized water were flowed through the PMR at an inlet pressure of 0.75 bar (position E in Figure S1a). The effective membrane area is 63.61 cm^2^, calculated based on four membranes within the PMR module. Figure S1b shows the flow direction of the solution in the membrane module. The precise thickness of the catalytic layer is difficult to quantify because the NM88B crystals exhibit integrated growth and are highly dispersed across the Al_2_O_3_ support surface (Figures S1b­(2 and 3)). Figure S1c shows the light irradiance spectra for a 400 W metal lamp (position F in Figure S1a).

### Characterization

Scanning electron microscopy (SEM; S-4800N, Hitachi) was used to characterize the morphologies and microstructures of the photocatalyst membranes and LDPE particles. Energy-dispersive spectrometry (EDS; 7900F, JEOL) was used to determine the elemental composition of the photocatalyst membranes. X-ray diffraction (XRD, Bruker D2 PHASER XRD) with Cu Kα radiation (λ = 1.5406 Å) was employed to examine the crystallinity. X-ray photoelectron spectroscopy (XPS; Thermo Fisher K-Alpha) was used to identify the oxidation states. Fourier transform infrared (FTIR; Tracer-100, Shimadzu) spectra were recorded to analyze the functional groups. A particle size and zeta potential analyzer (ELSZ-2000, Otsuka) was used to characterize the particle size distribution of LDPE particles via dynamic laser scattering (DLS). The optical properties of the samples were characterized via ultraviolet–visible spectroscopy (UV–vis, U-3900, Hitachi). Fe K-edge X-ray absorption fine structure (XAFS) spectra were measured at SPring-8 BL01B1 and KEK-PF BL-9A using the fluorescence method (sample) and transmission method (reference). N K-edge XAFS spectroscopy was performed at KEK-PF BL-7A using the fluorescence method. The photon energies of the Fe K-edge and N K-edge XAFS spectra were calibrated accordingly to previous studies, using the first peak position of the first derivative curve of the spectrum of Fe foil (7112.0 eV)^41^ and the first peak position of the spectrum of BN powder (401.0 eV)^42^ as reference points, respectively.

### Molecular Dynamics (MD) Simulations

Classical MD simulations were conducted using the LAMMPS package^43^ to investigate the intrinsic water permeation properties of the MIL-88B­(Fe) structure and the NH_2_-MIL-88B­(Fe). Figure S2 illustrates the simulation system, which consisted of the feed side, a slab of either structure, and the permeate side. Two graphene sheets were included as rigid pistons on both sides of the system to apply a transmembrane pressure. The slab structure with a thickness of ∼20 Å was cleaved by breaking the Fe–O bonds with a terminal surface parallel to the (100) plane. The resulting dangling bonds of Fe atoms were compensated by hydroxide (−OH) groups to ensure charge neutrality. We note that, despite the thin membrane, prior studies by some of us^44,45^ have shown that the water flux of the modeled membranes is linearly proportional to the reciprocal of the membranes’ thickness, and the computed flux can be extrapolated to membranes of experimentally relevant thickness. These indicate that the small thickness of ∼20 Å should be deemed sufficiently representative. Each structure was relaxed using the semiempirical PM7 method^46^ implemented in the MOPAC package.^47^

MD simulations included three main stages: (i) energy minimization to preliminary relax the systems, (ii) equilibrium MD (EMD) simulations to reach an equilibrium water density and to saturate the studied structures, and (iii) nonequilibrium MD (NEMD) simulations with an applied transmembrane pressure (i.e., ∼165 MPa) to probe the water permeation property. The applied pressure of ∼165 MPa, a value that is much higher than that applied in experimental and industrial RO processes, was employed to achieve more accurate sampling within a typical simulation time of tens of nanoseconds. As shown in Figure S3, the water flux was found to scale linearly with the applied pressure, suggesting that water permeability is insensitive to the applied pressure. In these calculations, intermolecular potentials were modeled using Lennard–Jones (LJ) and Coulombic interactions. The LJ parameters (i.e., δ and ε) were adopted from the DREIDING force field^48^ for the structural atoms, and their atomic charges were derived from the PM7 calculations. Water molecules were described by the SPC/E model^49^ with the SHAKE algorithm to maintain their rigidity. The Lorentz–Berthelot mixing rule was applied to estimate pairwise LJ parameters. All the adopted potential parameters are summarized in Tables S1 and S2.

It should be noted that, while employing bond-order-based force fields (e.g., reactive force field (ReaxFF)) may enable the investigation of dynamic degradation reaction of LDPE within photocatalytic membranes, a specifically parametrized set of potential parameters has not yet been developed. In addition, the corresponding simulation system may be enormous, making such calculations not trivial. As such, this study has opted to employ classical potential to first explore the permeation behavior of water through the two studied membranes without considering the reaction aspect.

## Results and Discussion

### Characterization of the M88B and NM88B Powders

Figure S4a and Figure S4b display the morphology and appearance of the M88B and NM88B powders, respectively revealing particles with a spindle shape and a more elongated spindle morphology. The XRD patterns of these samples (Figure S4c) show the characteristic peaks of M88B at 9.59, 10.75, 12.72, 16.73, and 19.05°, which correspond to the (101), (002), (102), (103), and (200) crystal planes, respectively. In the XRD pattern of NM88B, the peaks for the (101), (102), (103), and (200) planes are similar to those of M88B, but the peak for the (002) plane appears at a lower angle than that for the (101) plane, likely due to the more elongated spindle shape of NM88B revealed by the SEM observations.^4^ The FTIR spectra of M88B and NM88B (Figure S4d) show peaks for the presence of Fe–O (stretching in the Fe–O–Fe cluster) and Fe–O (μ_3_-O bridging oxygen) functional groups at 554 and 624 cm^–1^, respectively, along with peaks for C=O functional groups at 1393, 1600, and 1665 cm^–1^.^50^ In addition, the spectrum of NM88B exhibits distinct absorption bands for −NH_2_ groups at 3335 and 3470 cm^–1^,^51^ confirming the −NH_2_ modification.

Figure S5a shows the XAFS spectra of M88B and NM88B. The Fe K-edge X-ray absorption near edge structure (XANES) spectra of M88B, NM88B, and the reference samples FeO, γ-Fe_2_O_3_, α-Fe_2_O_3_, α-Fe_3_O_4_, α-FeOOH, β-FeOOH, and γ-FeOOH confirm the presence of Fe^3+^ coordination in M88B and NM88B.^52^ In the N K-edge XAFS spectrum of NM88B (Figure S5b), a pre-edge peak is observed at 400.9 eV, implying the presence of −NH_2_, as previously reported.^53^ Figure S5c shows that the Fe K-edge FT-extended X-ray absorption fine structure (EXAFS) spectra of M88B and NM88B display a peak for Fe–O bonds at 1.6 and 1.6 Å, respectively. The shift of the Fe–O bond peak in NM88B and the peak overlap due to close proximity suggests that the −NH_2_ modification introduced a distinct structural change. Figure S5d presents a schematic of the Fe vicinity in M88B and NM88B, indicating that the Fe–O bond in NM88B is shorter, positioning the Fe atom closer to the center, which contributes to the structural differences observed between the two materials.

Figure S6a illustrates the mass loss of LDPE particles after photodegradation using M88B and NM88B powders under light irradiation, revealing a 37.9% mass loss for NM88B and a 16.2% mass loss for M88B. This result demonstrates the superior photocatalytic activity of NM88B for LDPE degradation. Figure S6b presents the results of a scavenger test used to identify the reactive species in the photodegradation of LDPE over NM88B. The addition of 10 mM *p*-benzoquinone, isopropanol, EDTA-2Na, and potassium bromate as scavengers of •O_2_^–^, •OH, hole (h^+^), and electron (e^–^), respectively, all led to a decrease in photocatalytic activity. Notably, the sequestration of h^+^ resulted in the most significant suppression of weight loss, indicating that direct oxidation by holes is the dominant pathway for LDPE degradation. Furthermore, the similar inhibitory effects observed for (e^–^ and •O_2_^–^) suggest that electrons do not directly degrade the plastic but instead facilitate the process primarily through the reduction of O_2_ to generate •O_2_^–^. These findings confirm that while multiple ROS participate, the photocatalytic process is primarily driven by h^+^-mediated oxidation. To further corroborate the radical evolution, EPR spectroscopy was performed using DMPO and TEMP as spin traps (Figure S7). Although the raw spectra exhibited complex overlapping signals, likely due to DMPO self-decomposition or the formation of solvent-derived alkyl radicals,^54,55^ spectral deconvolution via EasySpin clearly resolved the characteristic 1:2:2:1 quartet signal of DMPO-•OH (Figure S7a,b). While both M88B and NM88B showed comparable •O_2_^–^ intensities, NM88B displayed a significantly enhanced signal for singlet oxygen (^1^O_2_), as shown in Figure S7c. According to the mechanism proposed by Nosaka et al., ^1^O_2_ can be generated via the reduction of triplet O_2_ to •O_2_^–^, followed by an oxidative process through hole (h^+^) capture.^56,57^ This pathway not only facilitates the production of high-energy ^1^O_2_ but also serves as an effective electron–hole separation mechanism. These EPR findings are highly consistent with the scavenger tests, reinforcing the dominant role of h^+^. Specifically, the scavenging of h^+^ not only prevents the direct oxidation of LDPE but also inhibits the oxidative transformation of •O_2_^–^ into ^1^O_2_, thereby leading to the substantial decrease in overall photocatalytic degradation efficiency.

### Characterization of the MRS-NM88B and MRS-M88B Membranes

To integrate the membranes in the PMR, M88B and NM88B were directly grown on the top of an alumina membrane. SEM and EDS analyses were conducted to examine the surface morphology and elemental distribution of the photocatalyst membrane before and after synthesis. As shown in Figure 1a, the SEM image of the alumina membrane reveals large, stacked alumina crystals. Figure 1b presents the SEM image of the MRS-M88B membrane, where spindle-shaped particles corresponding to MIL-88B­(Fe) crystals are clearly visible. Notably, the NM88B particles and their shapes are similar to those of M88B (Figure 1c), consistent with previous results.^58,59^ The EDS mapping in Figure 1d confirms that the alumina membrane primarily consists of Al and O elements, with trace amounts of C and Si. EDS mapping of the M88B and NM88B membranes (Figure 1e,f) further reveals the uniform distribution of Fe and N elements, in addition to the Al, C, O, and Si elements derived from the alumina substrate.^60,61^ These results confirm the successful deposition and incorporation of MIL-88B­(Fe) crystals on the alumina surface. Table 1 shows the EDS elemental ratios of each membrane. In the MRS-NM88B membrane, Fe and N elements are present with similar atomic percentages, whereas the MRS-M88B membrane exhibits only the Fe element. This result indicates that both NM88B and M88B are homogeneously coated on the alumina surface.

**Figure 1:**
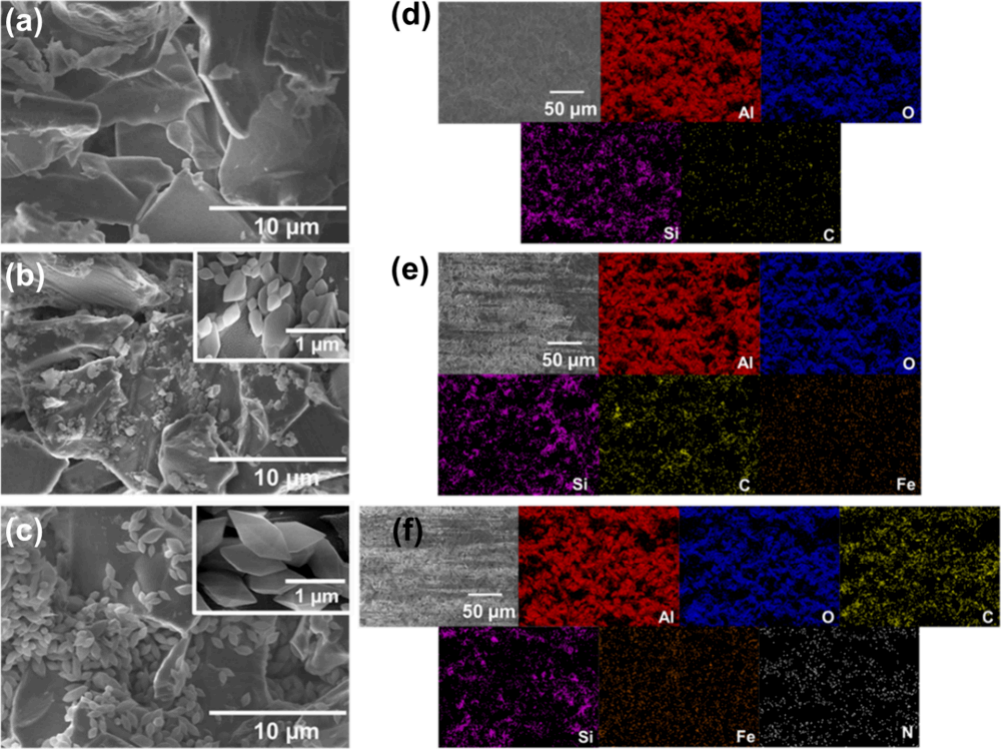
SEM images of the (a) alumina, (b) MRS-M88B, and (c) MRS-NM88B membranes. EDS mapping of the (d) alumina, (e) MRS-M88B, and (f) MRS-NM88B membranes.

**Table 1:** Element Atomic Percentages of the Alumina, MRS-NM88B, and MRS-M88B Membranes

	atomic ratio (%)					
	Fe	N	C	O	Al	Si
**alumina**	0.03	0.00	5.57	57.10	33.28	4.02
**MRS-NM88B**	1.57	1.34	29.68	44.05	21.45	1.91
**MRS-M88B**	0.72	0.00	26.29	46.92	21.45	2.99

Figure 2a illustrates the XRD patterns of MRS-NM88B and MRS-M88B after being scrapped off from the membrane surface. The primary diffraction peaks of MRS-M88B and MRS-NM88B at approximately 25, 36.6, 38, and 42° correspond to the (012), (104), (110), and (113) crystal planes of α-Al_2_O_3_.^62^ In addition, the XRD pattern of the MRS-M88B membrane exhibits the characteristic peaks of MIL-88B­(Fe) at 9.59, 10.75, 12.72, 16.73, and 19.05°, which correspond to the (101), (002), (102), (103), and (200) crystal planes (CCDC No. 647646). In contrast, the pattern of NM88B displays peaks corresponding to the (002), (101), (102), (103), and (202) planes at 9.2, 10.1, 12.8, 16.7, and 20.2°, respectively, which match the standard simulated peaks of NH_2_-MIL-88B­(Fe).^63,64^ In contrast with M88B, the peak for the (002) plane of NM88B appears at a lower diffraction angle than that for the (101) plane. This discrepancy is due to the preferred orientation growth of the spindle-shaped morphology, as observed in the SEM images.^59^ These findings confirm the structural differences between M88B and NM88B, which stem from the −NH_2_ modification. Figure 2b and its inset present the UV–vis spectra and digital images of the alumina, MRS-NM88B, and MRS-M88B membranes. The spectrum of the MRS-NM88B membrane exhibits an extended absorbance in the visible-light region, approximately between 500 and 800 nm, whereas the MRS-M88B membrane gives rise to a weaker absorbance in the range of 400–600 nm. The enhanced light absorption of the MRS-NM88B membrane can be attributed to the surface −NH_2_ modification, which aligns with previous findings.^59^ These results indicate that the MRS-NM88B membrane can be activated by visible light, suggesting its potential as an effective photocatalyst.

**Figure 2:**
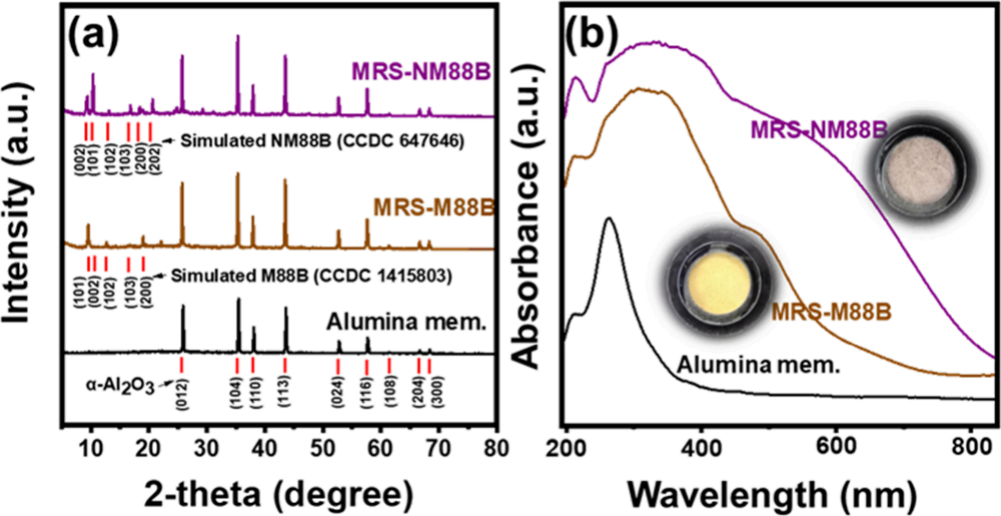
(a) XRD patterns and (b) UV–vis spectra of the alumina, MRS-M88B, and MRS-NM88B membranes.

Figure 3a shows the full-scan XPS spectra of the alumina, MRS-M88B, and MRS-NM88B membranes. The spectrum of the alumina membrane displays peaks corresponding to C 1s, O 1s, Al 2p, and Al 2s, which are in accord with its elemental composition. In the spectrum of the MRS-NM88B membrane, additional peaks associated with N 1s and Fe 2p are observed, whereas the spectrum of the MRS-M88B membrane shows only an additional peak for Fe 2p level. Figure 3b presents the Fe 2p spectra of the MRS-NM88B and MRS-M88B membranes, in which distinct signals corresponding to Fe 2p_3/2_, Fe 2p_1/2_, and Fe^3+^ appear at binding energies of 711.5, 725.5, and 729 eV, respectively.^65,66^ Notably, the Fe 2p_3/2_ peak of MRS-NM88B shifts toward a lower binding energy compared with that of MRS-M88B, indicating the formation of Fe–N coordination and an increased local electron density around the Fe centers. The C 1s spectra (Figure 3c) show signals at 711.5 and 725.5 eV, which can be assigned to C–C, C–O–C, and O–C=O bonds. Meanwhile, the N 1s spectrum of the MRS-NM88B membrane (Figure 3d) exhibits characteristic peaks for Fe–N, C–N, and −NH_2_ bonds at binding energies of 398.9, 400.05, and 401.9 eV, respectively.^61,67^ These results confirm the formation of MIL-88B­(Fe) in the MRS-M88B membrane, and the successful – NH_2_ modification of the MRS-NM88B membrane.

**Figure 3:**
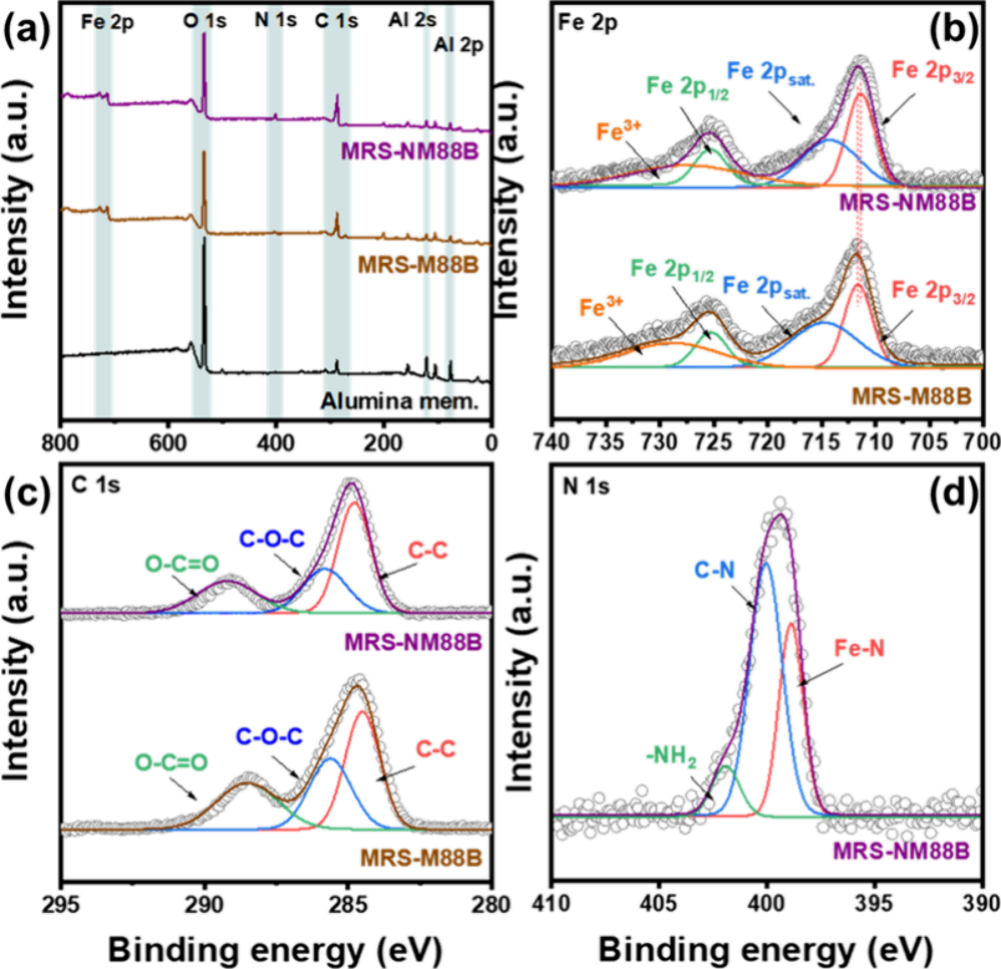
(a) Survey-scan XPS spectra of the alumina, MRS-88B, and MRS-NM88B membranes. High-resolution (b) Fe 2p and (c) C 1s XPS spectra of the MRS-M88B and MRS-NM88B membranes. (d) N 1s XPS spectrum of the MRS-NM88B membrane. Open circles denote the experimental data.

The water and glycerol contact angles of each membrane were dynamically measured to assess their hydrophilicity (Figure 4).^68^ As shown in Figure 4a, a water droplet could not maintain its shape for >0.5 s on the alumina surface, resulting in a water contact angle of 0°. In contrast, the MRS-M88B and MRS-NM88B membranes exhibits water contact angles of approximately 58.9 and 34.6°, respectively, at 0.5 s. Figure 4b presents the contact angle measurements using glycerol, which is more viscous and stable than water. The contact angle of glycerol on the alumina, MRS-NM88B, and MRS-M88B membranes are 25.1, 31.5, and 43.8°, respectively, at 10 s. Figure S8 shows the glycerol contact angle of the three membranes over time, revealing that the contact angle gradually decreased. The enhanced hydrophilicity of NM88B compared with M88B can be attributed to the presence of abundant surface −NH_2_ groups, as evidenced in Figure 3d.

**Figure 4:**
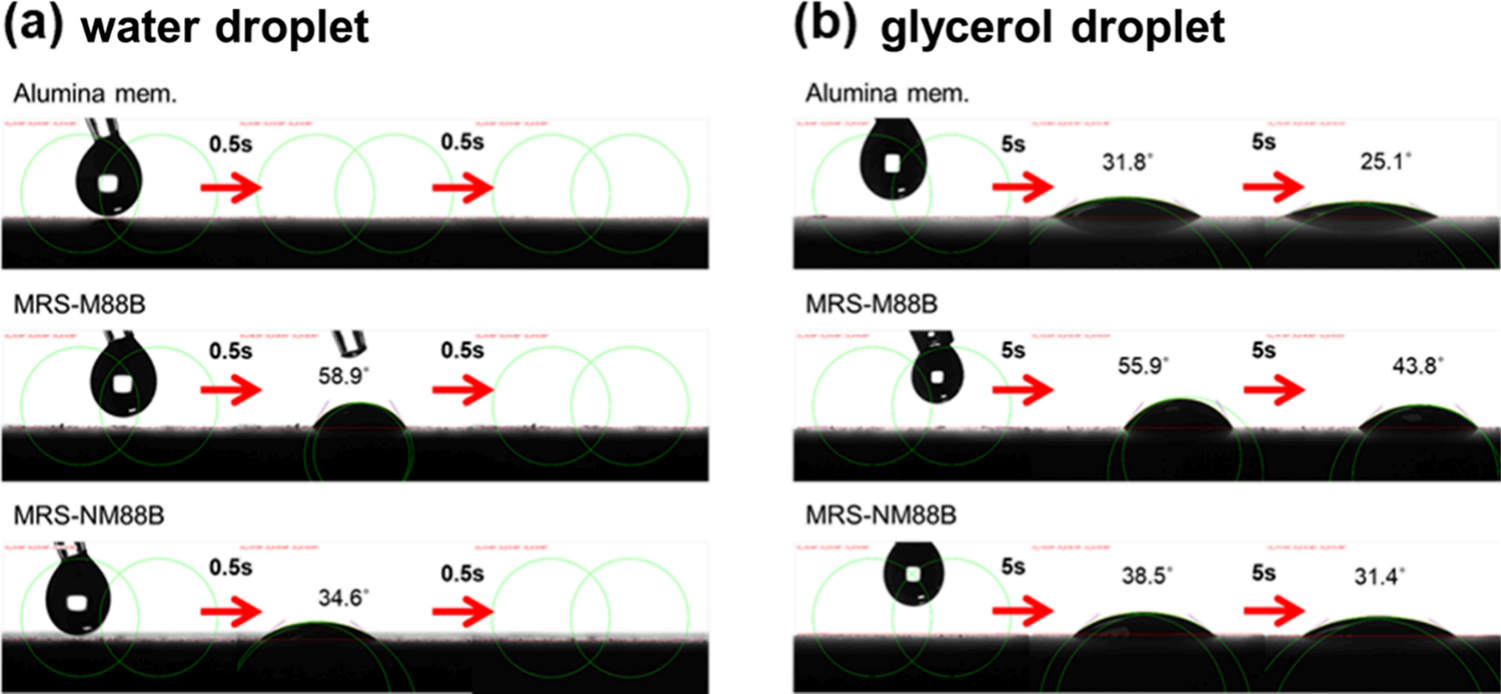
Contact angle measurements using (a) deionized water and (b) glycerol for the alumina, MRS-M88B, and MRS-NM88B membranes.

### LDPE Particle Removal Using the PMR with the MRS-NM88B and MRS-NM88B Membranes

Figure 5 shows the permeance flux of the photocatalytic membranes in the PMR for the removal of LDPE particles. The normalized permeance flux was also calculated to understand the relative differences between permeance under light irradiation and dark conditions. The MRS-NM88B and MRS-M88B membranes exhibit high initial permeance fluxes of 9225 and 8400 LMH·bar^–1^, respectively, under dark conditions. After 16 h of experimentation, the permeance fluxes of the MRS-NM88B and MRS-M88B membranes decrease to 8415 and 7348 LMH·bar^–1^, respectively, which correspond to normalized permeance fluxes of 0.91 and 0.88. Under light irradiation, the permeance fluxes of the MRS-NM88B and MRS-M88B membranes are initially 9435 and 8450 LMH·bar^–1^, respectively, and decrease to 8652 and 7490 LMH·bar^–1^ with normalized permeance fluxes of 0.92 and 0.89, respectively, after 16 h of irradiation. The permeance fluxes are slightly greater under light irradiation than under dark conditions, which indicates that the photocatalytic membrane can slightly reduce surface fouling under light irradiation. The permeance flux of these membranes is closely associated with their hydrophilicity. Thus, the alumina membrane exhibits the highest permeance under both dark and light conditions, with values of approximately 11,000 and 11,271 LMH·bar^–1^, respectively. Meanwhile, the MRS-M88B membrane, which has the largest water and glycerol contact angles, exhibits the lowest permeance flux among the tested membranes.

**Figure 5:**
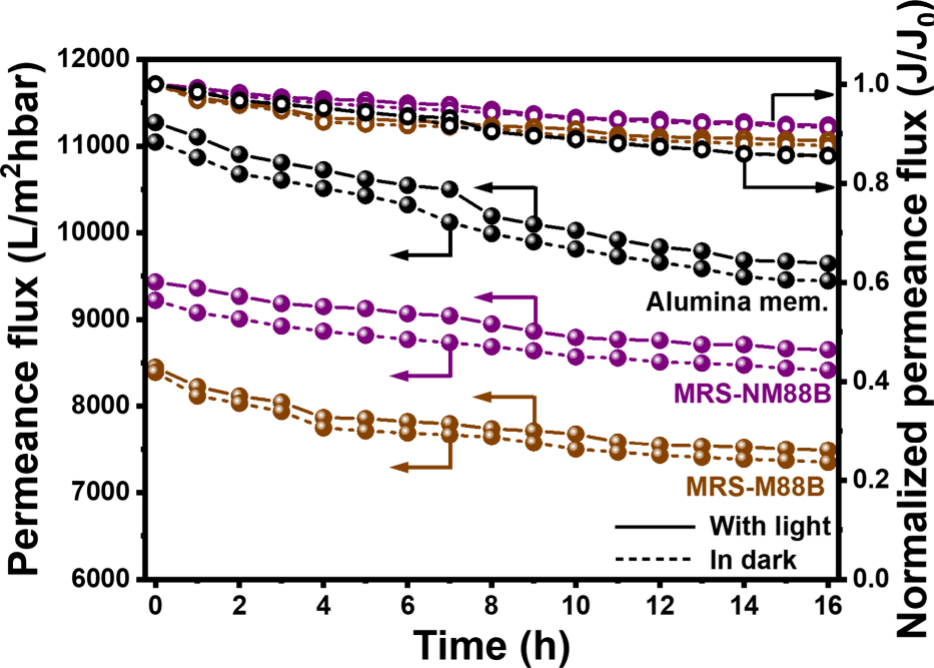
Permeance flux for LDPE removal in the PMR using the alumina, MRS-M88B, and MRS-NM88B membranes under dark (dashed line) and light (solid line) conditions (hollow points represent permeance flux; solid points represent normalized permeance flux; reaction conditions: deionized (DI) water = 1 L; [LDPE] = 25 mg/L; light source = 400 W metal lamp; light intensity = 180 W/m^2^; inlet pressure = 0.75 bar). All data are presented as the mean of three independent experiments.

Interestingly, distinct from the experimental observations, the MD-predicted permeation flux of the NH_2_-functionalized MIL-88B­(Fe) structure is slightly lower (Figure 6a), which can be attributed to the smaller pore limiting diameter (PLD) of the NH_2_-functionalized MIL-88B­(Fe) (5.64 Å) relative to the pristine MIL-88B­(Fe) (5.96 Å). These results suggest that not the MOF layer in the membranes but the support layer or their interfaces or interparticular voids play a dominant role in controlling the permeation flux. Notably, as shown in Figure 6b, the MD-calculated average water–MOF interaction along the permeation direction is enhanced for the NH_2_-functionalized MIL-88B­(Fe) structure compared with the unfunctionalized one. This finding agrees with the experimental observation that the former structure is relatively more hydrophilic. To this end, the MD simulation indicates that introducing NH_2_ groups into the MOF layer improves the overall hydrophilicity of the membrane, while the MOF layer is not the permeation bottleneck, ultimately leading to a higher flux for the NM88B membrane.

**Figure 6:**
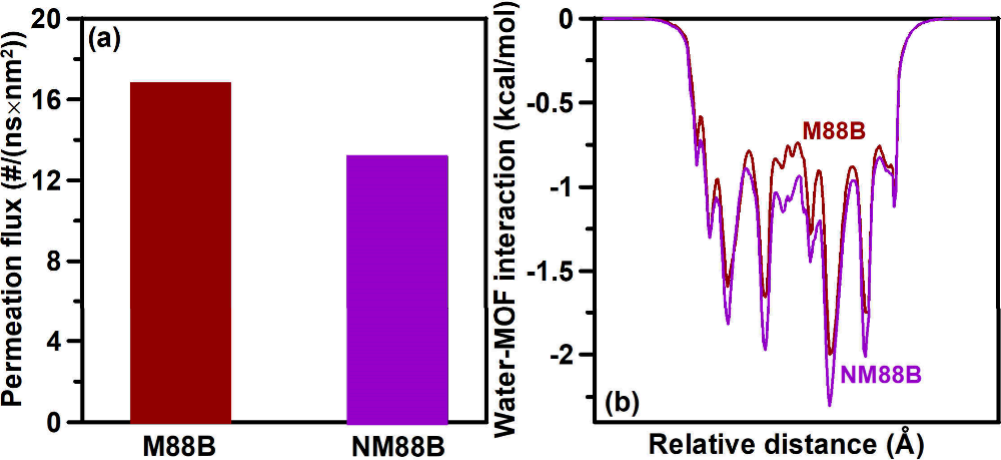
(a) MD-predicted permeation flux of MIL-88B­(Fe) (M88B) and NH_2_-functionalized MIL-88B­(Fe) (NM88B). (b) Average water–MOF interaction along the permeation direction across the M88B and NM88B layers. A negative energy value represents an attractive water–MOF interaction.

The photocatalytic and filtration performance of the PMR was determined by calculating the mass of LDPE particles using a mass balance approach. Equation 1 represents the mass balance under dark conditions, where the superscript D denotes dark. *M*_total_^D^ represents the initial mass of LDPE particles, and *M*_mem._^D^ and *M*_sys._^D^ correspond to the mass of LDPE particles retained on the membrane surface and accumulated in the PMR (including the tank and pipelines), respectively, at the end of the experiment. In addition, *M*_B._^D^ represents the mass of LDPE particles that were either degraded or not collected within the PMR. Equation 2 describes the mass balance under light conditions, where the superscript L indicates light. Accordingly, the photocatalytic degradation of LDPE can be evaluated using eq 3, where mp refers to the mass change of LDPE particles between light and dark conditions. This allows us to quantify the extent of photocatalytic degradation in the PMR.

In the dark:

$$M_{\mathrm{total}}^{D}=M_{\mathrm{mem}.}^{D}+M_{\mathrm{sys}.}^{D}+M_{B.}^{D}$$ 1

Under light irradiation:

$$M_{\mathrm{total}}^{L}=M_{\mathrm{mem}.}^{L}+M_{\mathrm{sys}.}^{L}+M_{B.}^{L}$$ 2

Mass difference of LDPE between light and dark conditions:

$$M_{B.}^{L}=M_{B.}^{D}+M_{P}$$ 3

Figure S9 shows the rejection ratio of LDPE on each membrane over time under dark conditions. The rejection ratio for all membranes gradually increased over time when filtering LDPE particles. This enhancement in the rejection ratio is mainly due to the increased filtration time and permeance flux, which enlarge the number of cycles in the circulation system. When using photocatalytic membranes, the presence of photocatalysts on the surface causes a decrease in permeance flux compared with alumina membranes. This results in a reduced number of cycles and a lower membrane rejection rate for the photocatalytic membranes (*M*_mem._^L^ and *M*_mem._^D^). However, owing to its better flux, the MRS-NM88B membrane has a higher membrane rejection rate than the MRS-M88B membrane. As shown in Figure 6a and Figure 6b, the *M*_mem._^L^ and *M*_mem._^D^ values of the alumina membrane are 92.7 and 90.3%, respectively, indicating its high rejection efficiency for LDPE particles. Notably, no obvious difference was observed between dark and light conditions, suggesting that the alumina membrane exerted a negligible photocatalytic effect. In contrast, the *M*_mem._^D^ values of the MRS-NM88B and MRS-M88B membranes are 80.8 and 73.0%, respectively, and the corresponding *M*_B._^D^ values are 16.1 and 17.7% (Figure 7a). This result indicates that the MRS-NM88B membrane demonstrated better LDPE rejection than the MRS-M88B membrane, likely due to its superior hydrophilicity and permeance flux. Meanwhile, the *M*_mem._^L^ values of the MRS-NM88B and MRS-M88B membranes are 59.7 and 53.6%, respectively, and the corresponding *M*_B._^L^ values are 38.4 and 35.7% under light conditions (Figure 7b). The rejection rates of both membranes for LDPE particles are lower under light irradiation than in the dark, which can be attributed to the photocatalytic degradation of LDPE particles. The photocatalytic performance of the MRS-NM88B and MRS-M88B membranes was further evaluated, as shown in Figure 7c. The mass loss (*M*_P_) due to photocatalysis was 22.3% for the MRS-NM88B membrane and 18.0% for the MRS-M88B membrane. These results suggest that both membranes effectively degraded LDPE particles under light irradiation, with the MRS-NM88B membrane exhibiting a higher photocatalytic efficiency.

**Figure 7:**
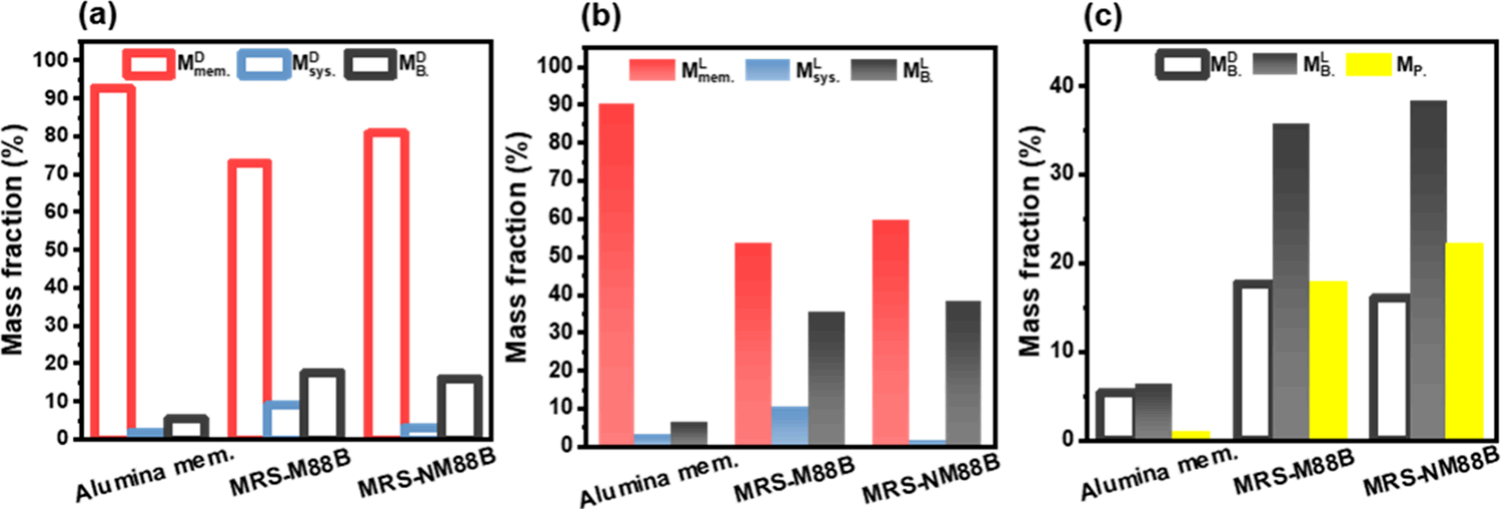
Mass fraction of LDPE particles in the PMR using the alumina, MRS-M88B, and MRS-NM88B membranes under (a) dark and (b) light conditions. (c) Evaluation of the photocatalytic LDPE degradation (reaction conditions: DI water = 1 L; [LDPE] = 25 mg/L; light source = 400 W metal lamp; light intensity = 180 W/m^2^; inlet pressure = 0.75 bar; time = 16 h). All data are presented as the mean of three independent experiments.

The LDPE particles were collected and characterized using SEM and DLS measurements (Figure 8). The SEM image of the original LDPE particles (Figure 8a) reveals nearly spherical particles with a regular, uniform shape and a larger particle size compared with those collected from the membranes after testing. In contrast, the LDPE particles collected from the MRS-M88B and MRS-NM88B membranes (hereinafter referred to as LDPE MRS-M88B and LDPE MRS-NM88B, respectively) after PMR tests exhibits a less complete, fragmented appearance with visibly smaller particle sizes (Figure 8b,c). Figure 8d shows the particle size distribution of these LDPE samples. Both LDPE MRS-NM88B and LDPE MRS-M88B particles exhibit a reduced particle size compared with the original LDPE particles, indicating that photocatalytic degradation contributed to the breakdown of LDPE particles during the PMR process.

**Figure 8:**
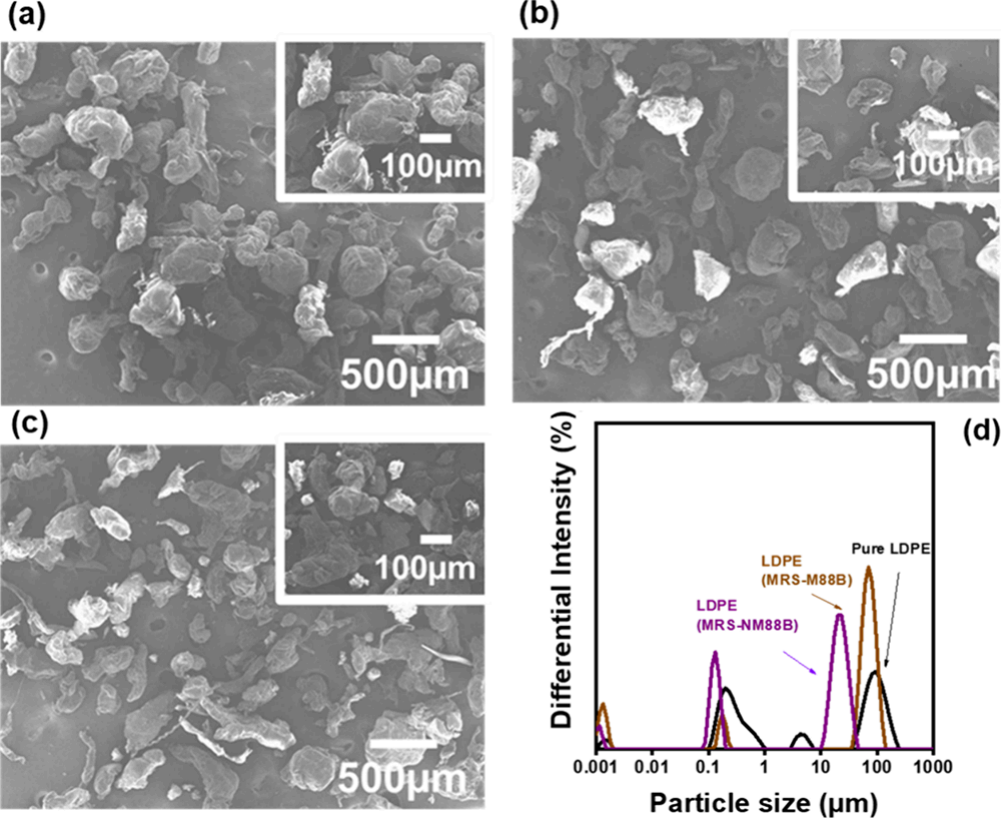
SEM images of (a) original LDPE particles and LDPE particles collected from (b) the MRS-M88B membrane surface (LDPE MRS-M88B) and (c) the MRS-NM88B membrane surface (LDPE MRS-NM88B). (d) Particle size distribution of these LDPE particles.

Figure 9 shows the FTIR spectra of the original LDPE particles and the LDPE MRS-M88B and LDPE MRS-NM88B particles. The spectrum of the original LDPE particles exhibits C–H bond rocking, bending, and stretching at 720, 1382 and 1465, and 2850 and 2922 cm^–1^, respectively.^69^ Meanwhile, the spectra of the LDPE MRS-NM88B and LDPE MRS-M88B particles show a decrease in the peak intensity for C–H bond bending, stretching, and rocking and the appearance of a peak at 1630 cm^–1^, indicating the formation of C=O and C=C bonds. This suggests that the degradation of LDPE particles by the photocatalytic membrane proceeds via the Norrish type II reaction, that is, the attack of C–H bonds by photocatalytically generated oxygenated radicals.^32^ The spectrum of the LDPE MRS-NM88B particles shows additional groups related to peroxy and free radicals between 1100 and 1380 cm^–1^, indicating that the photocatalytic degradation of LDPE particles in the MRS-NM88B membrane occurred to a considerable extent.^31^ The FTIR spectra of LDPE particles in the tank of the PMR after light exposure (Figure S10) reveal that the degree of oxidation of LDPE particles in the tank is lower compared with that of the LDPE particles rejected on the membrane, which suggests that prolonged light exposure is advantageous for improving the LDPE degradation. We also observed that the evolution of CO_2_ detected via GC confirms the potential for complete LDPE mineralization (Figure S11a). Although no specific liquid products were identified by HPLC, the significant increase in TOC values (Figure S11b) verifies the accumulation of dissolved organic intermediates, supporting the photocatalytic degradation of LDPE. These findings demonstrate that the photocatalytic process effectively drives LDPE from surface oxidation to final mineralization into CO_2_.

**Figure 9:**
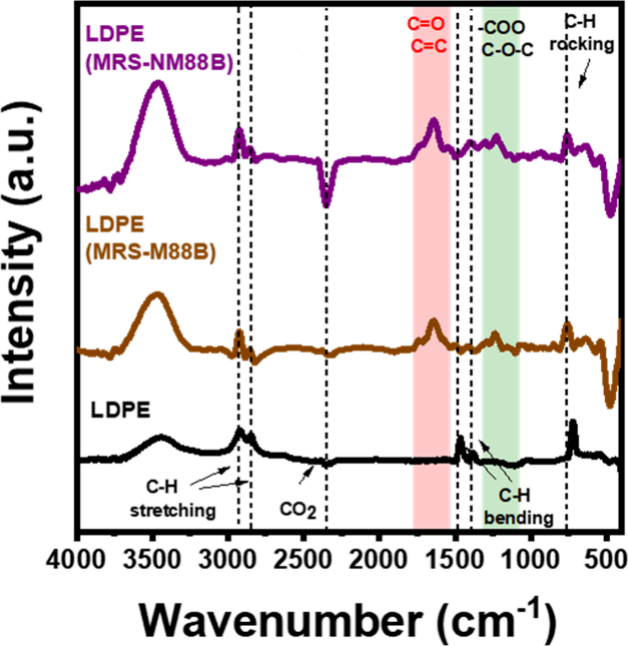
FTIR spectra of original LDPE particles and LDPE particles collected from the MRS-M88B membrane surface (LDPE MRS-M88B) and from the MRS-NM88B membrane surface (LDPE MRS-NM88B).

### Stability Experiment of the PMR Using the MRS-NM88B Membrane

Figure 10a illustrates the permeance flux for LDPE degradation using the MRS-NM88B membrane in the PMR over three consecutive cycles. The permeance flux of the MRS-NM88B membrane considerably decreases to 5404 LMH·bar^–1^ within the first 3 h in Run #2 (Figure 10a) and further stabilized between 3500 and 2900 LMH·bar^–1^ during the second and third cycles. The SEM image (Figure 10b) displays the spindle-like structure of NH_2_-modified MIL-88B­(Fe) on the surface of the MRS-NM88B membrane. Debris was observed on the membrane surface, likely from residual LDPE remaining after the PMR test. The contact angle of the NM88B membrane increased to approximately 130° (Figure 10c), indicating that the membrane became more hydrophobic after the PMR reaction. Elemental analysis (Figure 10d) shows a reduction in the Fe and N content after the PMR test, suggesting that −NH_2_ groups were partially leached from the surface during the reaction (Table 2). Nevertheless, the membrane structure and photocatalytic activity remain satisfactory after three cycles.

**Figure 10:**
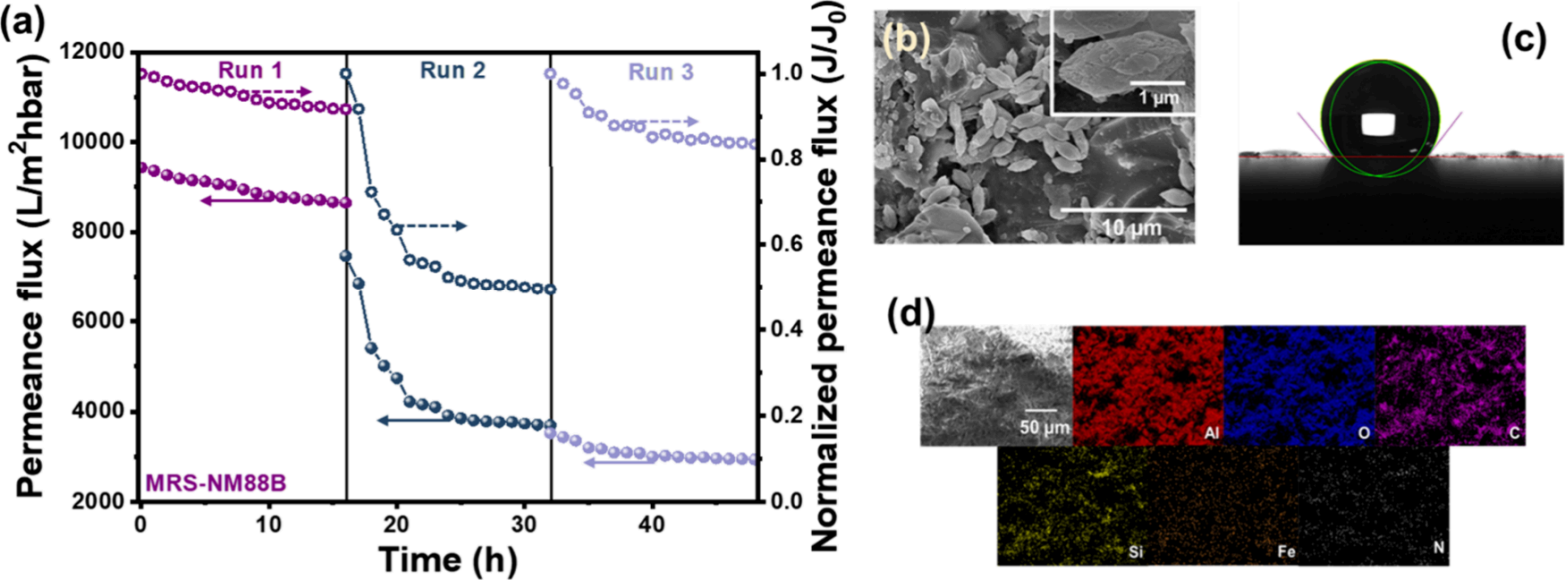
(a) Permeance flux during a three-run stability test of the PMR using the MRS-NM88B membrane for LDPE removal under light condition (hollow points represent permeance flux; solid points represent normalized permeance flux). (b) SEM image, (c) water contact angle, and (d) SEM-EDS mapping images of the MRS-M88B membrane after the three-run stability test (reaction conditions: DI water = 1 L; [LDPE] = 25 mg/L; light source = 400 W metal lamp; light intensity = 180 W/m^2^; inlet pressure = 0.75 bar).

**Table 2:** Element Atomic Ratios of the MRS-M88B Membrane after the Stability Test

	atomic ratio (%)					
	Fe	N	C	O	Al	Si
**before**	1.57	1.34	29.68	44.05	21.45	1.91
**after**	0.64	0.65	23.55	49.21	21.87	4.08

Figure S12a illustrates the LDPE removal efficiency of the PMR using the MRS-NM88B membrane over three consecutive runs. The degradation efficiency decreases during the third run, which can be attributed to sluggish photocatalytic kinetics and LDPE accumulation on the membrane surface, potentially shielding the membrane from light irradiation. Figure S12b presents the FTIR spectra of LDPE particles collected from the MRS-NM88B membrane during the cycle tests. The spectra reveal the formation of C=O and C=C bonds, supporting the occurrence of the Norrish type II reaction. However, the reaction rate in the third run was slower than that in the first run, suggesting a gradual decrease in the oxidation degree of LDPE particles, which is consistent with the Norrish type I reaction mechanism. Figure S12c displays the particle size distribution of LDPE particles after each cycle, showing a reduction in size after the photocatalytic reaction. Notably, the particle size is larger in the third run than in the first and second runs, correlating with the slower reaction rate observed in Figure S12b. This indicates that the photocatalytic activity decreased over time, possibly due to surface fouling or reduced light penetration. However, when the PMR was operated continuously for over 96 h (Figure S12d), the normalized permeance flux remained stable. A slight reduction in the absolute permeance flux was observed at 48 h, which was primarily attributed to the installation of a new plastic tube during the experimental setup rather than membrane fouling. Moreover, Figure S12e illustrates the mass fraction of LDPE degradation after 96 h, where the *M*_P_ value increased to approximately 36.32% over the extended duration. These results collectively confirm that our PMR system possesses excellent durability and maintains stable performance during long-term continuous operation, outperforming its behavior in discrete cycle operations. Furthermore, XRD analysis of the catalyst after the 96 h operation (Figure S12f,g) shows that the primary diffraction peaks remain identifiable despite a reduction in intensity. This suggests that while the MOF configurations may undergo partial structural modification or a decrease in crystallinity during the long-term reaction, the catalytic active sites remain sufficiently robust. Thus, the NM88B-PMR system maintains high efficiency even as the material undergoes these minor structural evolutions.

### Simulation of PMR for Treating LDPE Particles in Realistic Environments

To better understand the performance of the PMR in the treatment of daily domestic water and natural river water, river water was collected from the Keelung riverside in Taipei city. Figure 11 shows the permeance flux of the PMR with the MRS-NM88B membrane using deionized water and river water containing LDPE particles. River water containing impurities caused a more pronounced decrease in flux compared with deionized water. Interestingly, the extent of flux reduction when treating river water is greatly mitigated under illumination. The initial permeance flux in the dark is similar between river water and deionized water (8500–9200 LMH·bar^–1^). However, the flux using river water considerably drops to about 2500 LMH·bar^–1^ within 4 h and eventually declines to 1250 LMH·bar^–1^ after 16 h of operation in the dark. Under illumination, the permeance flux drops by about 54%, ultimately declining to 2145 LMH·bar^–1^ after 16 h. It is evident that illumination considerably mitigated the flux decline due to the photocatalytic degradation on the MRS-NM88B membrane surface, thereby preventing membrane fouling.

**Figure 11:**
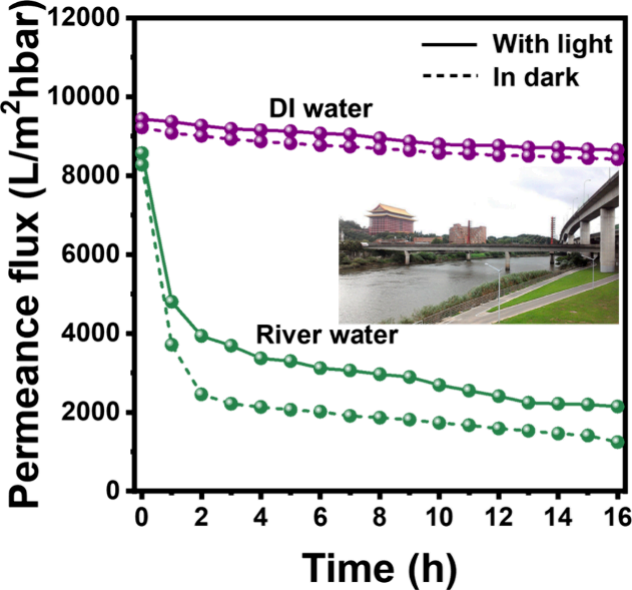
Permeance flux of the PMR with the MRS-NM88B membrane using DI water and river water containing LDPE particles. The inset shows the spot along the Keelung River nearby the Grand Hotel Taipei in Taiwan where river water was collected (reaction conditions: river water = 1 L; [LDPE] = 25 mg/L; light source = 400 W metal lamp; light intensity = 180 W/m^2^; inlet pressure = 0.75 bar).

Figure 12 presents the EDS mapping images at different magnifications of the river water filtrate before and after the PMR treatment. Prior to the PMR treatment, the river water contained various elements forming solid particles, as indicated by the SEM-EDS mapping. Notably, after the PMR process, these solid particles and associated compounds were completely removed. Table 3 shows the elemental analysis results of the river water before and after the PMR treatment. The results demonstrate that all contaminants were effectively removed, highlighting the efficacy of the PMR for pollutant remediation in real river waters samples and its potential for practical water purification in natural water bodies.

**Figure 12:**
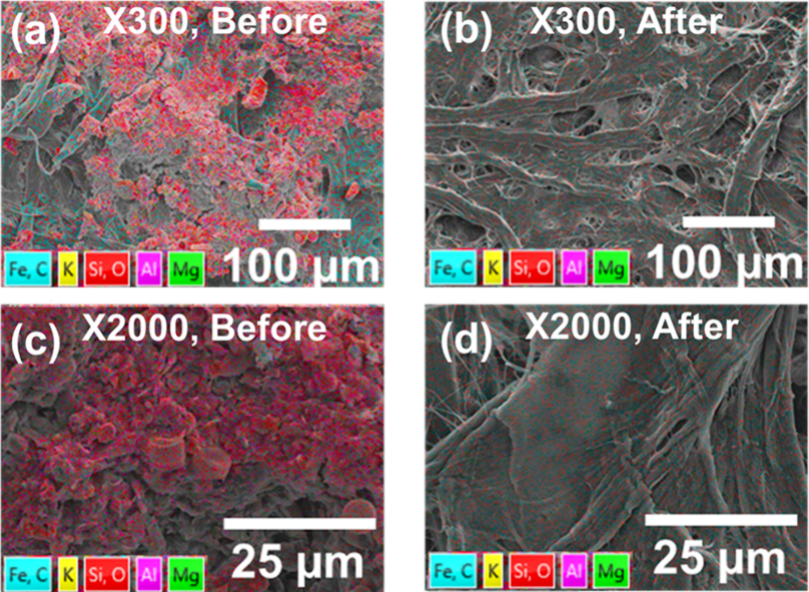
SEM-EDS mapping images at different magnifications of river water (a, c) before and (b, d) after treatment using the PMR with MRS-NM88B membrane (red, Si and O; pink, Al; blue, Fe and C; yellow, K; green, Mg).

**Table 3:** EDS Spectra of Elemental Weight Percentages in River Water

		element (wt. %)									
		O	C	Si	Fe	Al	K	P	Mg	Ca	S
×300 (magnification)	before	40.00	29.04	13.74	6.70	6.29	1.96	0.75	0.62	0.50	0.41
	after	51.6	47.8	0	0	0	0	0.3	0	0.2	0.1
×2000 (magnification)	before	38.4	10.1	22.9	10.2	12.5	3.1	1.2	1.1	0	0.4
	after	51.1	48.1	0	0	0	0.1	0.2	0	0	0.1

## Conclusions

This study presents a PMR integrating an NH_2_-modified MIL-88B­(Fe) membrane (NM88B) for the photocatalytic LDPE removal via filtration and degradation. The PMR with the NM88B membrane demonstrates improved hydrophilicity, higher permeance flux, and better photocatalytic performance compared with the PMR integrating the unmodified MIL-88B­(Fe) membrane. An LDPE particle removal efficiency of 96.9% in the dark and 22.3% mass loss under light irradiation are achieved using the PMR with the NM88B membrane. In addition, the PMR was successfully applied to treat river water containing LDPE. Our findings demonstrate the potential of the PMR integrating the NH_2_-modified MIL-88B­(Fe) membrane for LDPE removal and wastewater treatment.

## Supplementary Material



## Notes

Schematic illustration of the PMR system (Figure S1); schematic of a simulation system (Figure S2); water flux of the membranes as a function of applied pressures (Figure S3); SEM, XRD, and FTIR profiles (Figure S4); XANES and XAFS (Figure S5); photocatalytic LDPE degradation in a batch system and trapping tests (Figure S6); EPR tests (Figure S7); glycerol contact angles (Figure S8); rejection rate of the membrane (Figure S9); FTIR spectra of LDPE before and after the reaction (Figure S10); CO_2_ production in photocatalytic LDPE degradation and TOC test (Figure S11); recycle tests, long-term stability of PMR, and FTIR spectra and particle size of LDPE (Figure S12); nonbonded potential parameters (Table S1); and geometric features of water molecules (Table S2) (PDF)
