# Supplementary material for: Hydrophilicity-Enhanced NH2‑MIL-88B(Fe) Integrated Photocatalytic Membrane Reactor for Simultaneous Rejection and Degradation of Low-Density Polyethylene in Water Matrices
Source: ACS Appl Mater Interfaces. 2026 Mar 16;18(12):17688–99. doi: 10.1021/acsami.5c21187 (PMC13051431; doi:10.1021/acsami.5c21187)
Supplement: Supplementary file 1 [file am5c21187_si_001.pdf]

Supporting Information

Hydrophilicity-Enhanced NH_2-MIL-88B(Fe) Integrated Photocatalytic Membrane Reactor for Simultaneous Rejection and Degradation of Low-Density Polyethylene in Water Matrices

Guan-Yu Chen[a], Yu-Lin Chen[a], Kazuki Harada[b], Masaaki Yoshida[b], Qiang Lyu[c], Chia-Her Lin[d], Li-Chiang Lin[e], Chechia Hu[a*]

[a] Department of Chemical Engineering, Sustainable Electrochemical Energy Development Center (SEED), National Taiwan University of Science and Technology, Daan Dist., Taipei City, Taiwan 106

[b] Applied Chemistry, Graduate School of Sciences and Technology for Innovation, Yamaguchi University, Ube, Yamaguchi, Japan 755-8611

[c] Department of Materials Physics, China University of Petroleum (East China), Qingdao, Shandong 266580, China

[d] Department of Chemistry, National Tsing Hua University, Hsinchu City, Taiwan 300044

[e] Department of Chemical Engineering, National Taiwan University, Daan Dist., Taipei City, Taiwan 106

** To whom correspondence should be addressed:*
E-mail: chechia@mail.ntust.edu.tw (Prof. Chechia Hu)
Tel: 886-2-27376638; Fax: 886-2-27376644

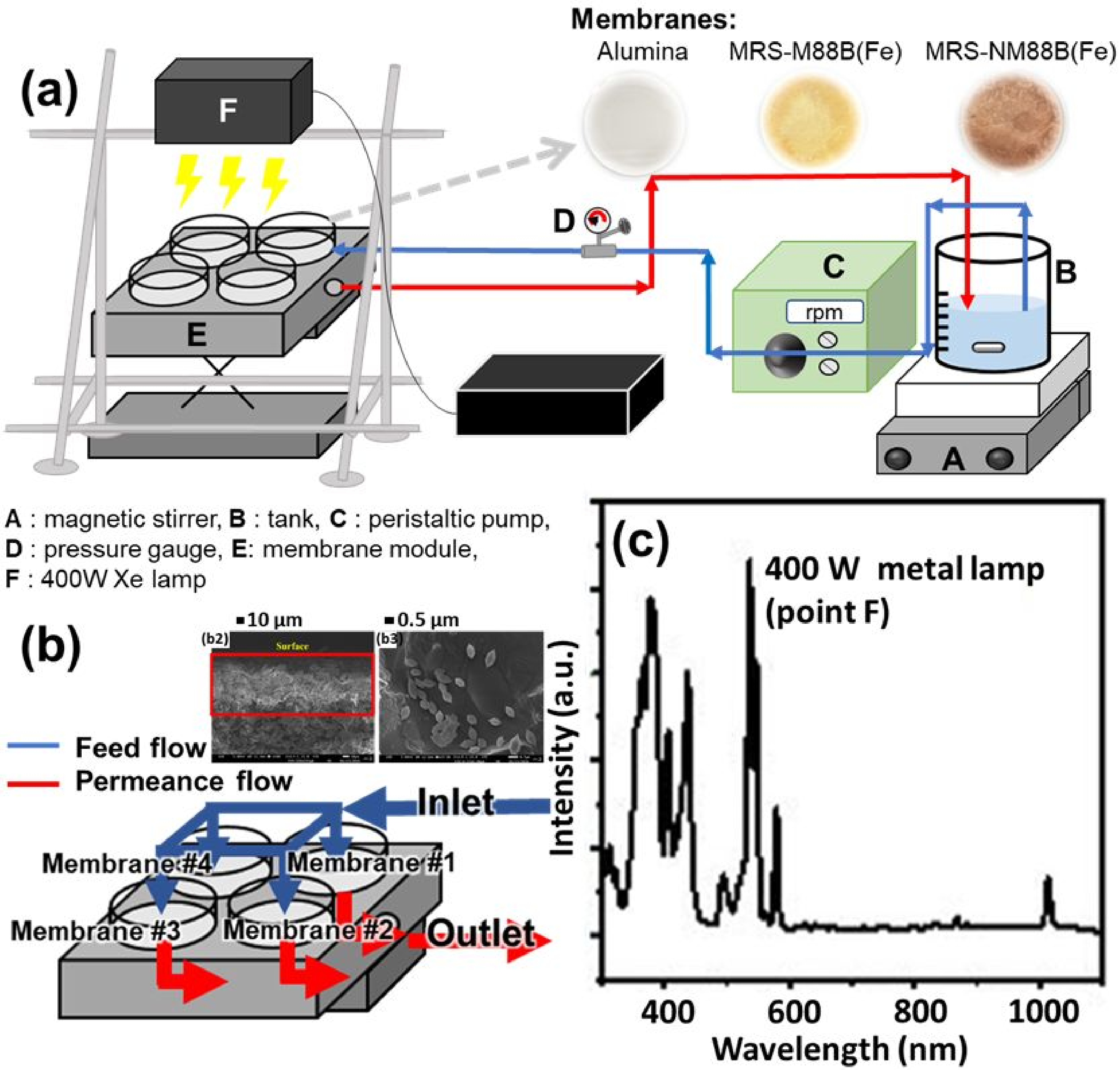

Figure S1 (a) PMR system (A: magnetic stirrer, B: tank, C: pump, D: pressure gauge, E: membrane modulus, F: 400 W Xe lamp; red, yellow and green lines represent feed flow, retentate flow, and permeance flow, respectively); (b) water flow inside the membrane modulus setup for series and parallel configurations; (c) light irradiance spectra for the xenon lamp. The insets in (b) show the cross-sectional SEM images of the MRS-NM88B membrane at different magnifications: (b2) 0.5 k and (b3) 15 k.

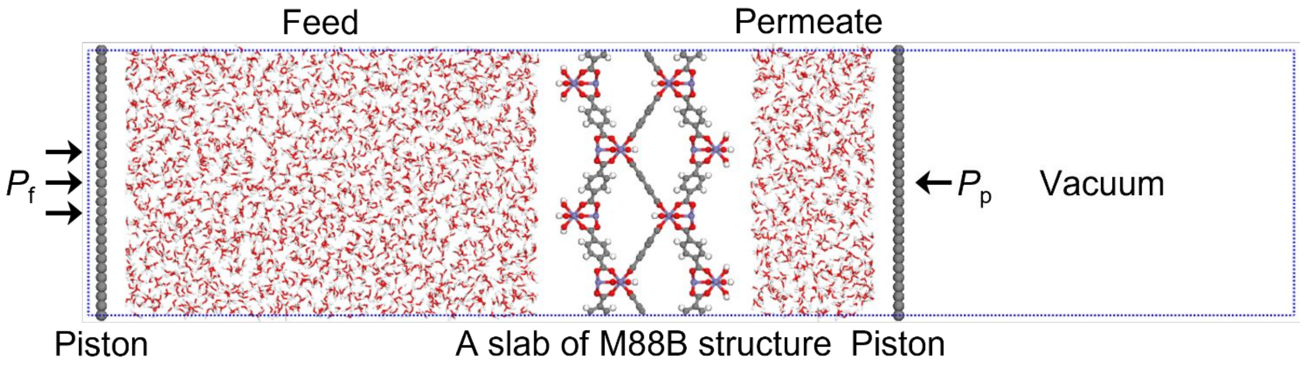

Figure S2 Schematic of a simulation system. A slab of MIL-88B(Fe) structure is used as the active separation layer for illustrative purposes, and two graphene pistons are used to apply a transmembrane pressure (i.e., $\Delta P = P_f - P_p$). Color codes: carbon, gray; hydrogen, white; oxygen, red; and iron, violet.

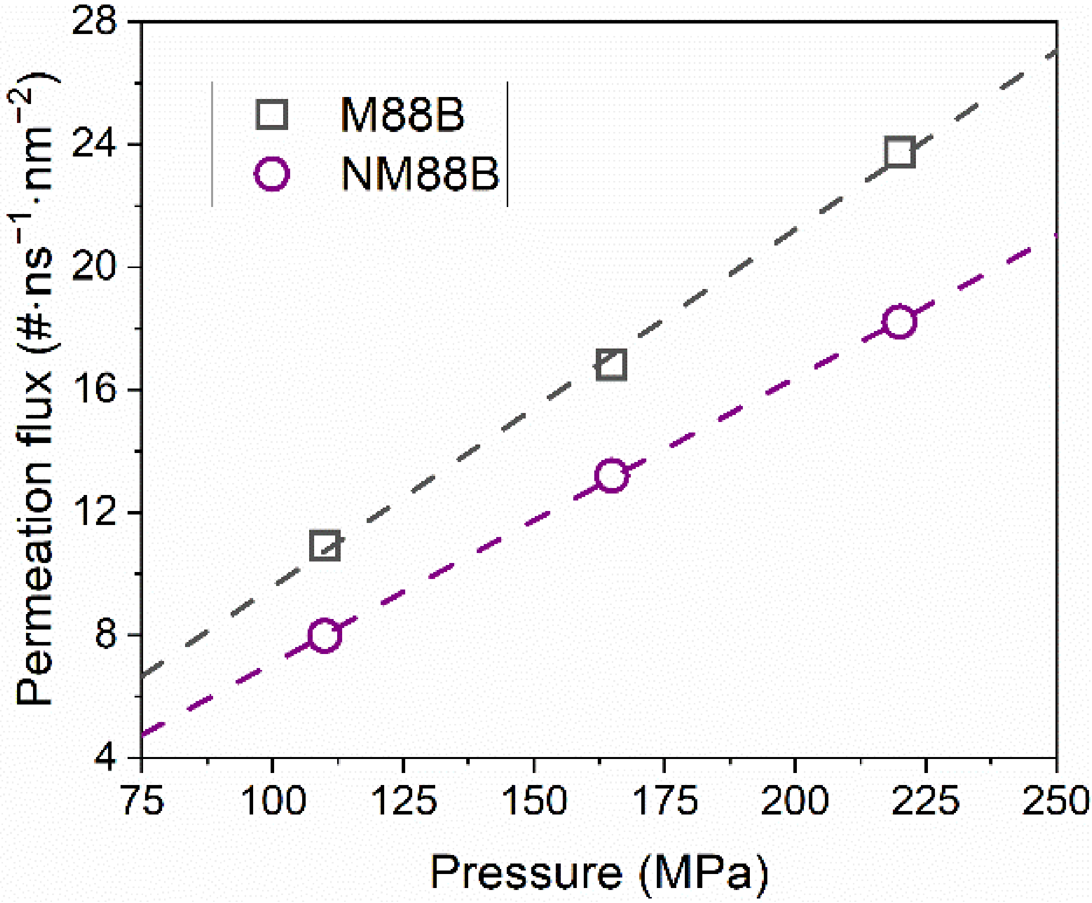

Figure S3 Water flux of M88B and NM88B membranes as a function of applied pressures.

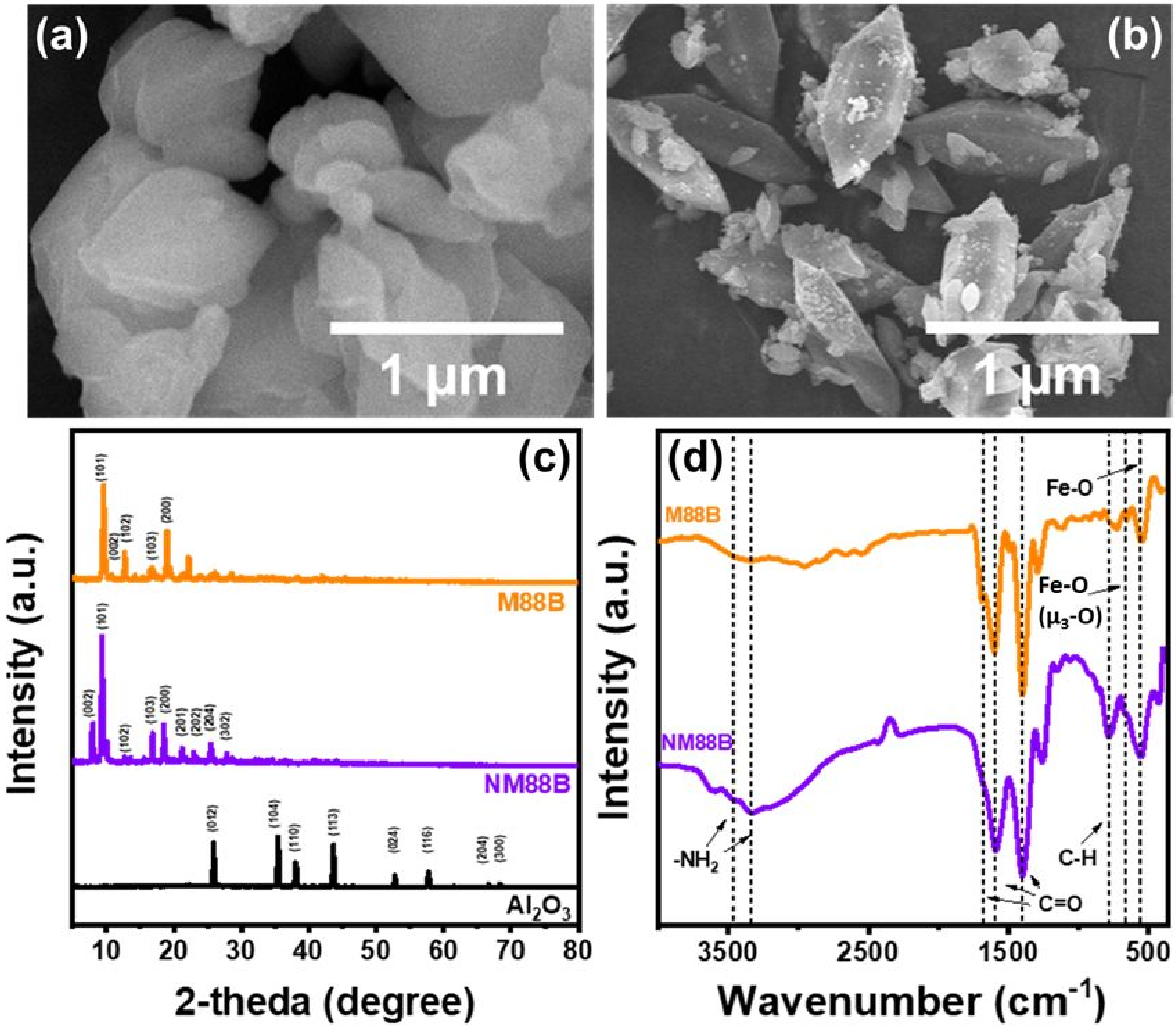

Figure S4 SEM images of (a) M88B and (b) NM88B. (c) XRD patterns and (d) FTIR spectra of M88B and NM88B.

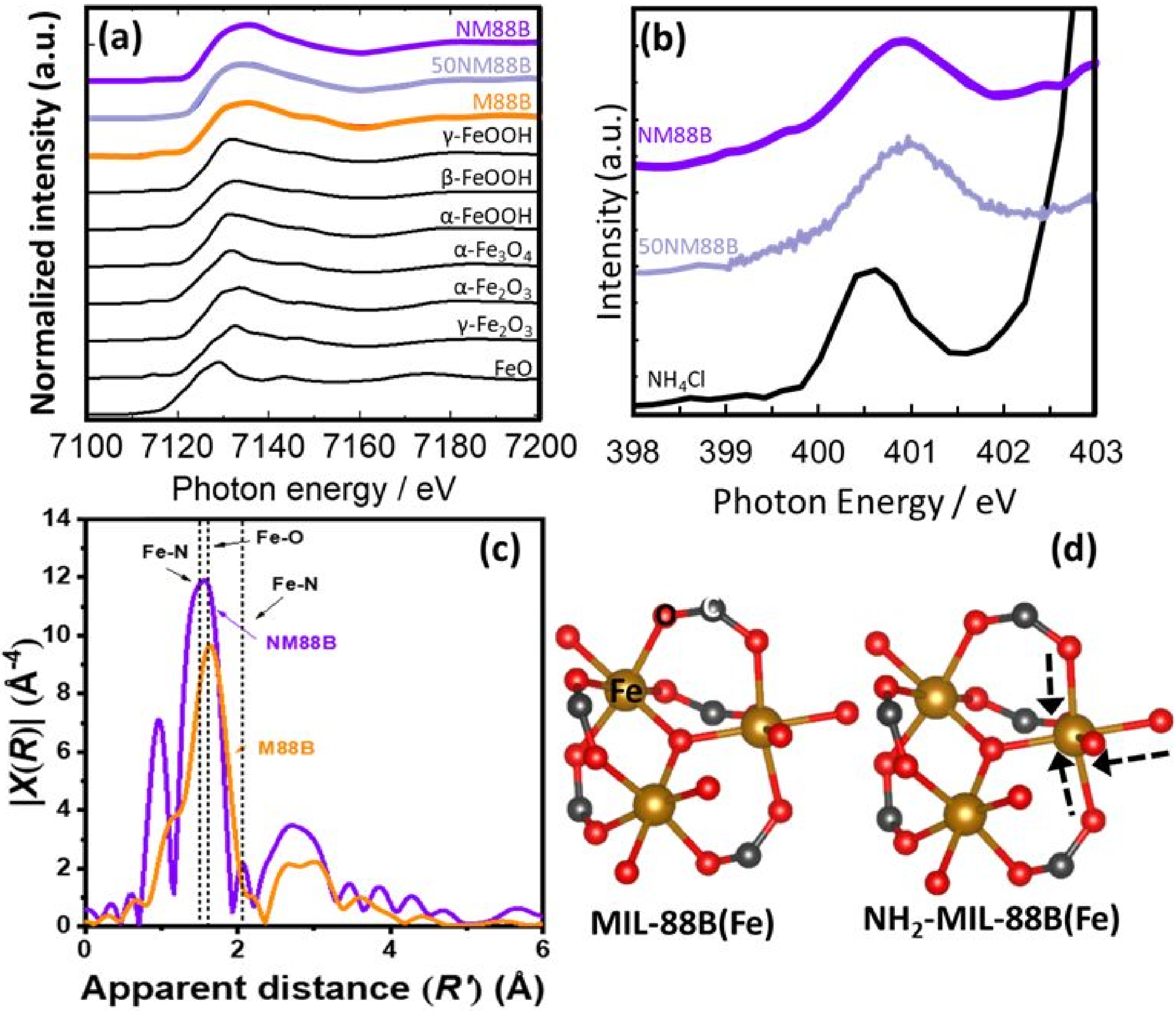

Figure S5 (a) Fe K-edge XANES spectra, (b) N K-edge XAFS spectra, (c) Fe K-edge FT-EXAFS spectra, and (d) schematic of M88B and NM88B.

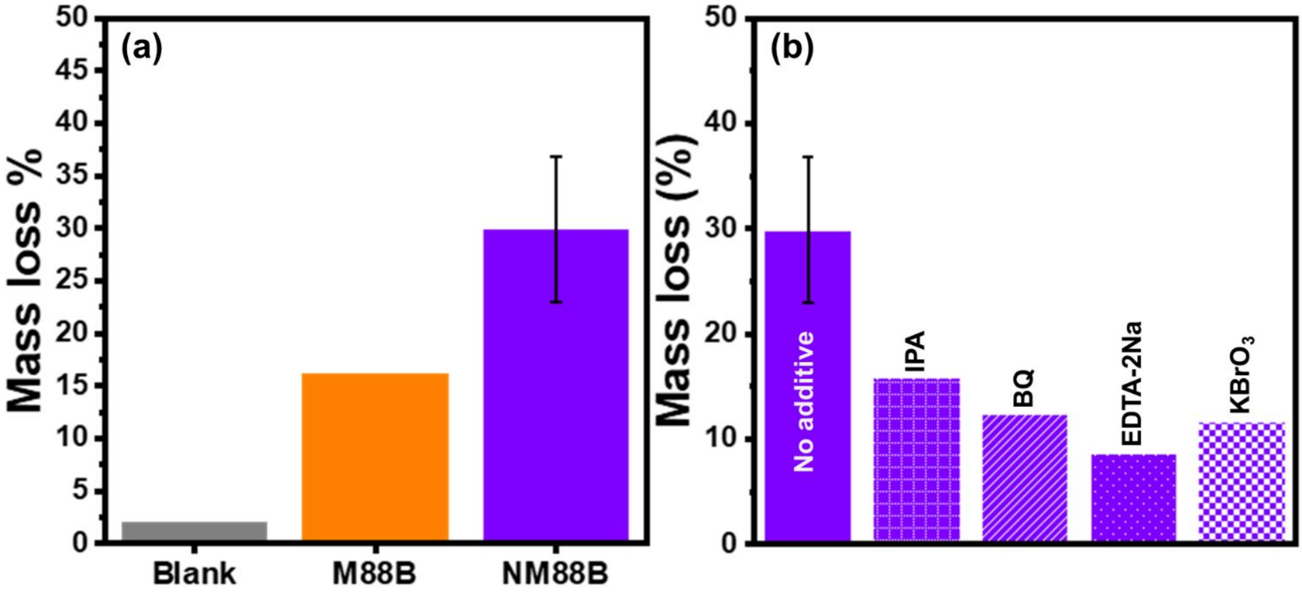

Figure S6 (a) Batch photodegradation experiments of LDPE particles with M88B and NM88B; (b) trapping reagent experiments using the NM88B photocatalyst (LDPE particles: 50 mg; catalyst: 20 mg; DI water: 50 mL; [trapping agent] =10 mM; light source: 400W Xe lamp; light intensity: 80 W/m^2; time: 16 h).

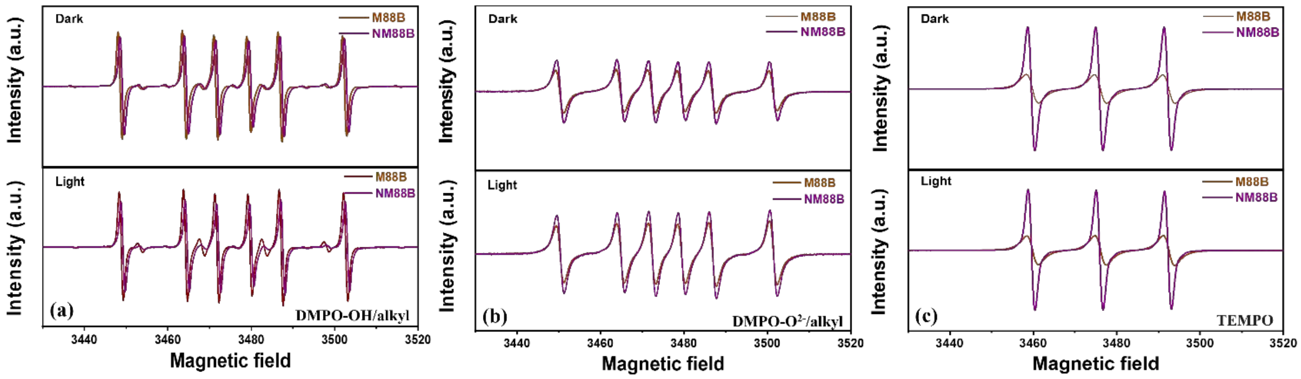

Figure S7 EPR spectra of M88B and NM88B using (a)DMPO+H_2O, (b)DMPO+MeOH, (c) TEMP+MeOH as trapping agent/solvent under dark and light irradiation to detect •OH, $•O_2^-$, and $^1O_2$, respectively.

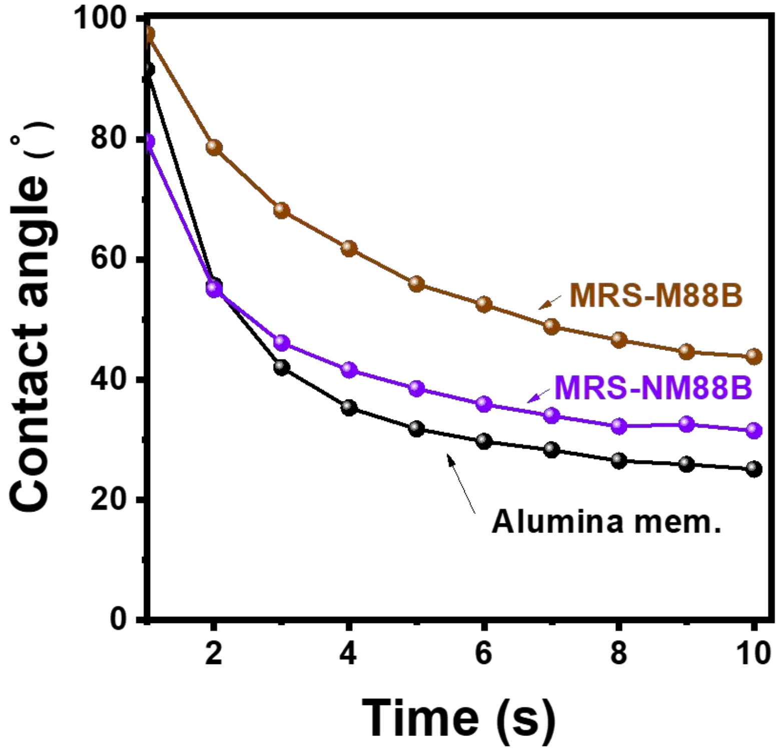

Figure S8 Glycerol contact angles on the aluminum, MRS-M88B, and MRS-NM88B membranes.

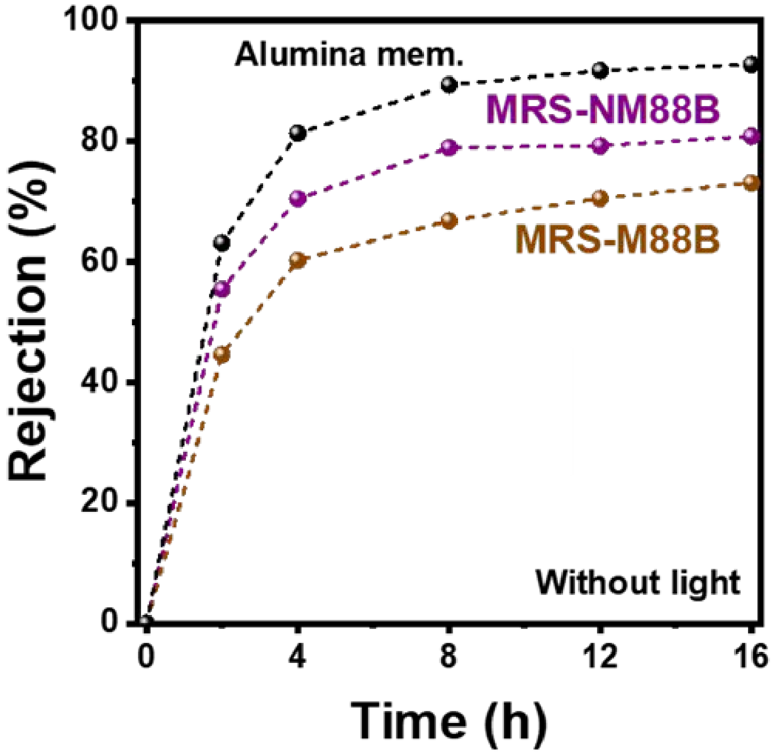

Figure S9 Change in the rejection rate of the membrane over time.

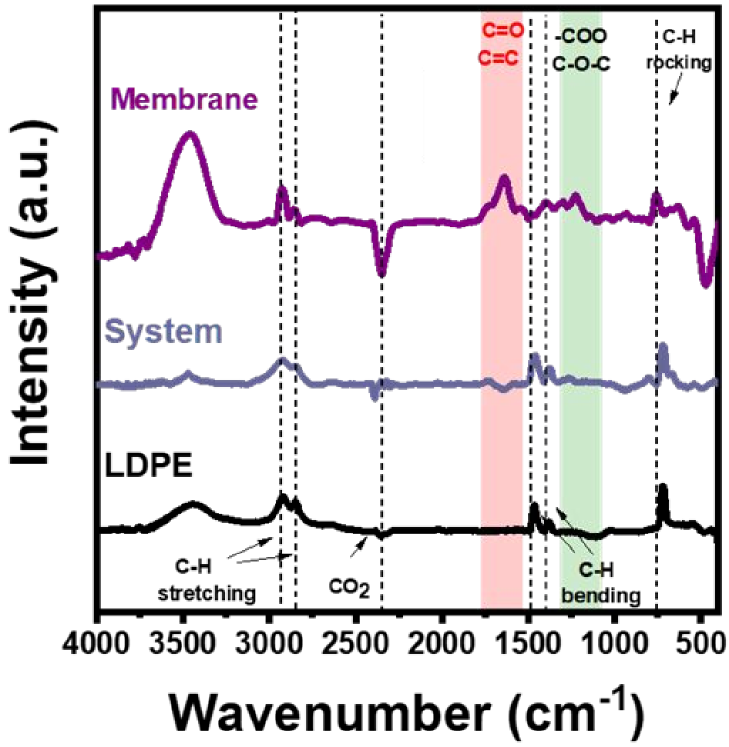

Figure S10 FTIR spectra of the original LDPE before the PMR test (LDPE) and the LDPE collected on the membrane (Membrane) and the system (System) after the PMR test.

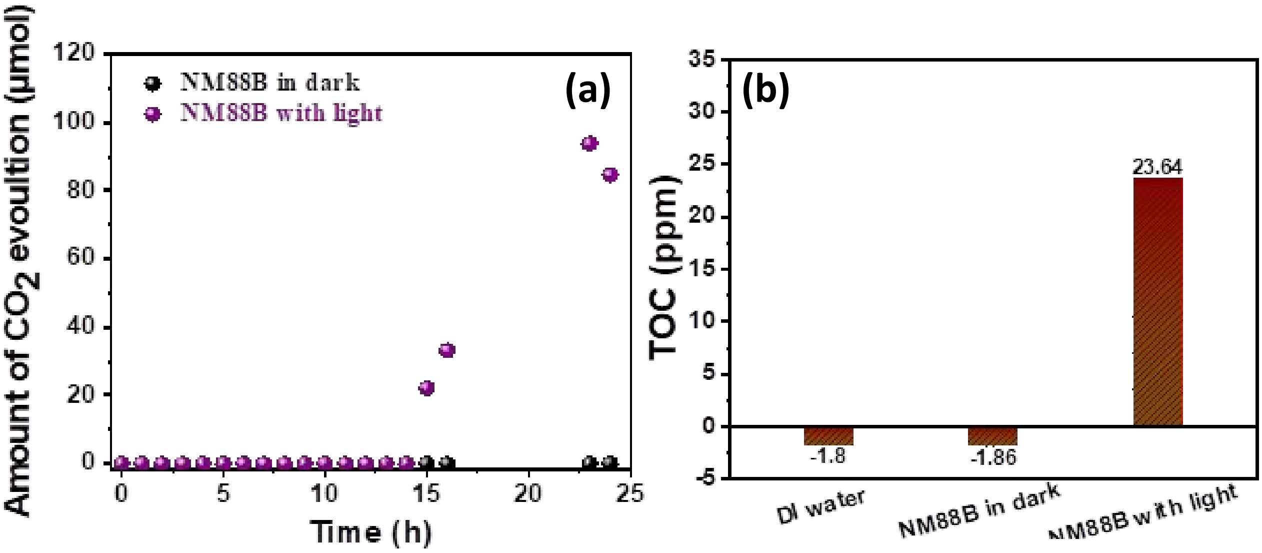

Figure S11 (a) CO_2 generation in photocatalytic degradation LDPE experiments, and the corresponding (b) total organic carbon values in DI water using NM88B in dark and light irradiation. (Reaction conditions: Lamp source = 400W metal lamp; Light intensity = 80 W/m^2; atmosphere = O_2; time = 24 hours; Deionized water = 100 mL; [LDPE] = 500 mg; [Catalyst] = 50 mg)

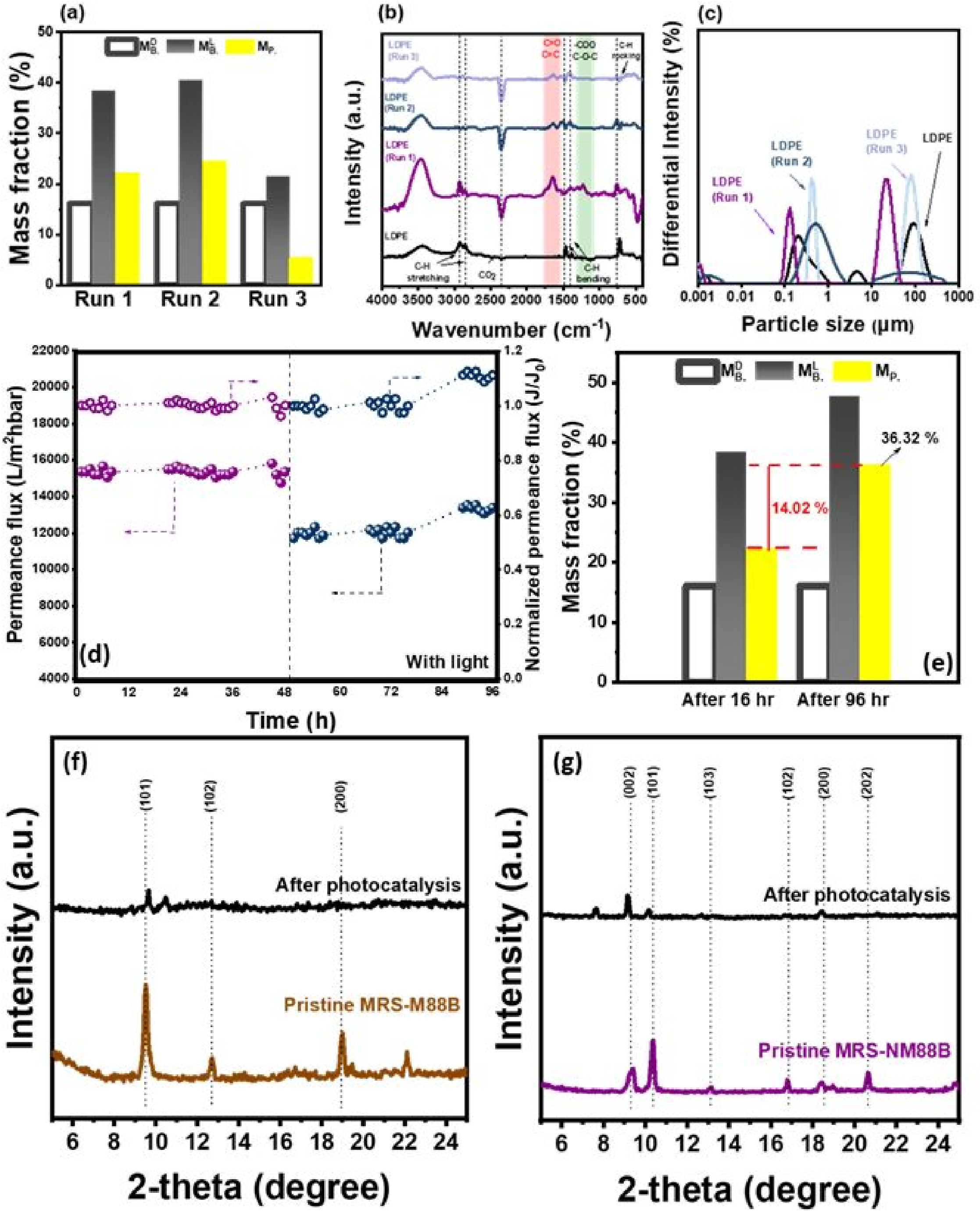

Figure S12 (a) Recycle tests in the PMR for LDPE removal. (b) FTIR spectra and (c) particle distribution of LDPE particles rejected in different recycle tests in the PMR. (d) Permeance and its normalized values in continuous long-term operation of PMR using the MRS-NM88B membrane for LDPE removal under light condition (hollow points represent permeance flux; solid points represent normalized permeance flux). (Reaction conditions: DI water = 1 L; [LDPE] = 25 mg/L; light source = 400 W metal lamp; light intensity = 180 W/m^2; inlet pressure = 0.75 bar). (e) Mass fraction of LDPE particles in a 96-h continuous operation in a PMR system using MRS-NM88B membranes to evaluate photocatalytic LDPE degradation. (Reaction conditions: DI water = 1 L; [LDPE] = 25 mg/L; light source = 400 W metal lamp; light intensity = 180 W/m^2; inlet pressure = 0.75 bar), (f,g) XRD patterns of MRS-M88B and MRS-NM88B membrane before and after the photocatalytic reactions.

Table S1 Non-bonded potential parameters for MIL-88B(Fe) (M88B) and NH_2-modified MIL-88B(Fe) (NM88B), water molecules, and graphene pistons. Van der Waals and electrostatic interactions are described by Lennard-Jones and Coulombic potentials, respectively (i.e., $U_{ij} = 4\varepsilon\ [(\sigma_{ij} / r_{ij})^{12} - (\sigma_{ij} / r_{ij})^{6}]$ and $V_{ij} = q_i q_j / (4\pi\varepsilon_0 r_{ij})$).

	**Atom type**	**ε (kcal·mol^{-1})**	**σ (Å)**	**q (e)**
**MIL-88B(Fe) / NH_2-modified MIL-88B(Fe)**	**H**	0.0152	2.8464	Atomic charges are derived from the PM7 calculations.
	**C**	0.0951	3.4730	
	**N**	0.0774	3.2626	
	**O**	0.0957	3.0332	
	**Fe**	0.0550	4.0447	
**Water molecules**	**O**	0.1553	3.1660	−0.8476
	**H**	0.0000	1.0000	0.4238
**Graphene Pistons**	**C**	0.0565	3.2140	0.0000

Table S2 Geometric features of water molecules described by the rigid SPC/E water model, with r_0 and θ_0 representing bond length and bond angle, respectively.

	r_0 (Å)	θ_0 (°)
**O−H**	1.00	
**H−O−H**		109.47
